# The oncogene protein kinase PIM1 regulates mammalian erythroblast enucleation

**DOI:** 10.1038/s42003-025-08869-0

**Published:** 2025-10-15

**Authors:** Huan Zhang, Yuanlin Xu, Yudong Li, Jingxin Zhang, Jingbo Xiao, Chenghui Wang, Yaoyao Wang, Xiuli An, Shijie Zhang

**Affiliations:** 1 School of Life Sciences, Zhengzhou University, Zhengzhou, China; 2 Department of Internal Medicine, the Affiliated Cancer Hospital of Zhengzhou University & Henan Cancer Hospital, Zhengzhou, China; 3 Laboratory of Membrane Biology, New York Blood Center, New York, NY USA

**Keywords:** Erythropoiesis, Differentiation

## Abstract

Erythroblast enucleation is a unique process during mammalian erythropoiesis, yet its regulatory mechanisms remain largely elusive. Here, we demonstrate the specific regulatory role of the oncogene PIM1, the most highly expressed protein kinase in orthochromatic erythroblasts, in enucleation. Unlike its well-established roles in cancer cell proliferation and survival, knockdown of PIM1 in human erythroid cells does not affect cell growth or apoptosis, but specifically inhibits erythroblast enucleation without altering differentiation. To elucidate the functional conservation of PIM1 in mammalian erythropoiesis, we generate *Pim1*^*fl/fl*^*EpoR*^*Cre*^ mice in which Pim1 is deleted in erythroid cells. Consistent with human erythropoiesis, deletion of Pim1 in mice has no detectable effect on apoptosis or differentiation of erythroid cells, but specifically inhibits erythroblast enucleation. Phosphoproteomic analysis reveals that PIM1 deficiency causes a pronounced decrease in phosphorylation of GTPase-associated proteins involved in actin assembly and vesicle trafficking. Functionally, this perturbation results in an aberrant distribution of F-actin and endocytic vesicles within enucleating cells. These findings reveal the unexpected role of PIM1 in normal erythropoiesis and enhance our understanding of mammalian erythroblast enucleation.

## Abstract (web summary)

The oncogene PIM1 regulates erythroblast enucleation via GTPase-dependent cytoskeletal remodeling and vesicle trafficking, independent of its canonical roles in cell proliferation and apoptosis.

## Introduction

Red blood cells, the most common type of blood cells in living organisms, are highly specialized for gas exchange in the lungs and peripheral tissues due to the abundant hemoglobin^1^. During erythropoiesis, hematopoietic stem cells undergo lineage specific commitment, generating erythroid progenitor cells: burst-forming units-erythroid (BFU-E) and colony-forming units-erythroid (CFU-E). CFU-E undergo 3 to 5 cellular division events to sequentially generate terminal differentiated erythroblasts: proerythroblasts (Pro), basophilic erythroblasts (Baso), polychromatic erythroblasts (Poly) and orthochromatic erythroblasts (Ortho)^2–4^. Enucleation, the process by which Ortho extrudes the nucleus to produce a nascent reticulocyte rich in cytoplasm and hemoglobin, along with a membrane-bound nucleus surrounded by a thin rim of cytoplasm (the pyrenocyte), is unique to mammals^5^. Despite being discovered for more than a century, the molecular mechanisms underlying this distinctive feature remain incompletely understood.

Erythroblast enucleation is a complex, multi-stage event in mammalian erythroid terminal differentiation, including nuclear condensation, polarization, extrusion, and final separation^6^. Proper nuclear condensation requires caspase-mediated nuclear opening^7–9^ and histone deacetylation during terminal erythropoiesis^10–13^. Subsequently, the condensed nucleus migrates to one side of the cell for polarization under the control of AURKA-regulated unipolar spindle constituted by microtubules^14–16^. Remodeling of the cell membrane, especially the asymmetric distribution of membrane proteins like ECT2^14^, provides an asymmetric contractility crucial for the extrusion of the nucleus. Thereafter, F-actin and myosin IIB form a contractile actin ring in the cleavage furrow to accomplish the final separation between the pyrenocyte and the reticulocyte^16–21^. Endocytic vesicles move and fuse at the division plane to facilitate erythroblast enucleation^22,23^. Numerous proteins have been demonstrated to be crucial for the process of enucleation. However, most of them are involved in downstream functional roles. The identity of upstream regulatory proteins in erythroblast enucleation remains largely unclear.

In eukaryotic organisms, reversible protein phosphorylation, catalyzed by protein kinases, is known to regulate the majority of cellular pathways, especially those involved in signal transduction. Although only approximately 1–2% of predicted genes encode protein kinases (about 560) in human, about 30% of cellular proteins are phosphorylated^24,25^. Many of them play critical roles in various growth, developmental processes, and responses to environmental stimuli. Whether protein kinase-mediated phosphorylation plays important roles in regulating erythroblast enucleation remains an open question.

According to the database of the gene-annotation portal BioGPS^26^, Bloodspot^27^ and our preliminary data, erythroblasts, especially Ortho, highly express the serine/threonine kinase PIM1, but so far, the role of PIM1 in regulating erythropoiesis or erythroblast enucleation has not been studied. In the present study, we found that PIM1 specifically regulated erythroblast enucleation in both human and mice without affecting cell proliferation or apoptosis, unlike its role in cancer. Mechanistically, PIM1 regulates erythroblast enucleation by controlling the assembly of F-actin and the distribution of endocytic vesicles via GTPase-related pathways. Our results thus identify a previously unknown regulatory factor for enucleation, unveil a novel function of the protein kinase PIM1, and provide mechanistic insights into mammalian erythroblast enucleation.

## Results

### PIM1 is highly expressed in human and mouse erythroblasts and upregulated in Ortho

**Fig. 1:**
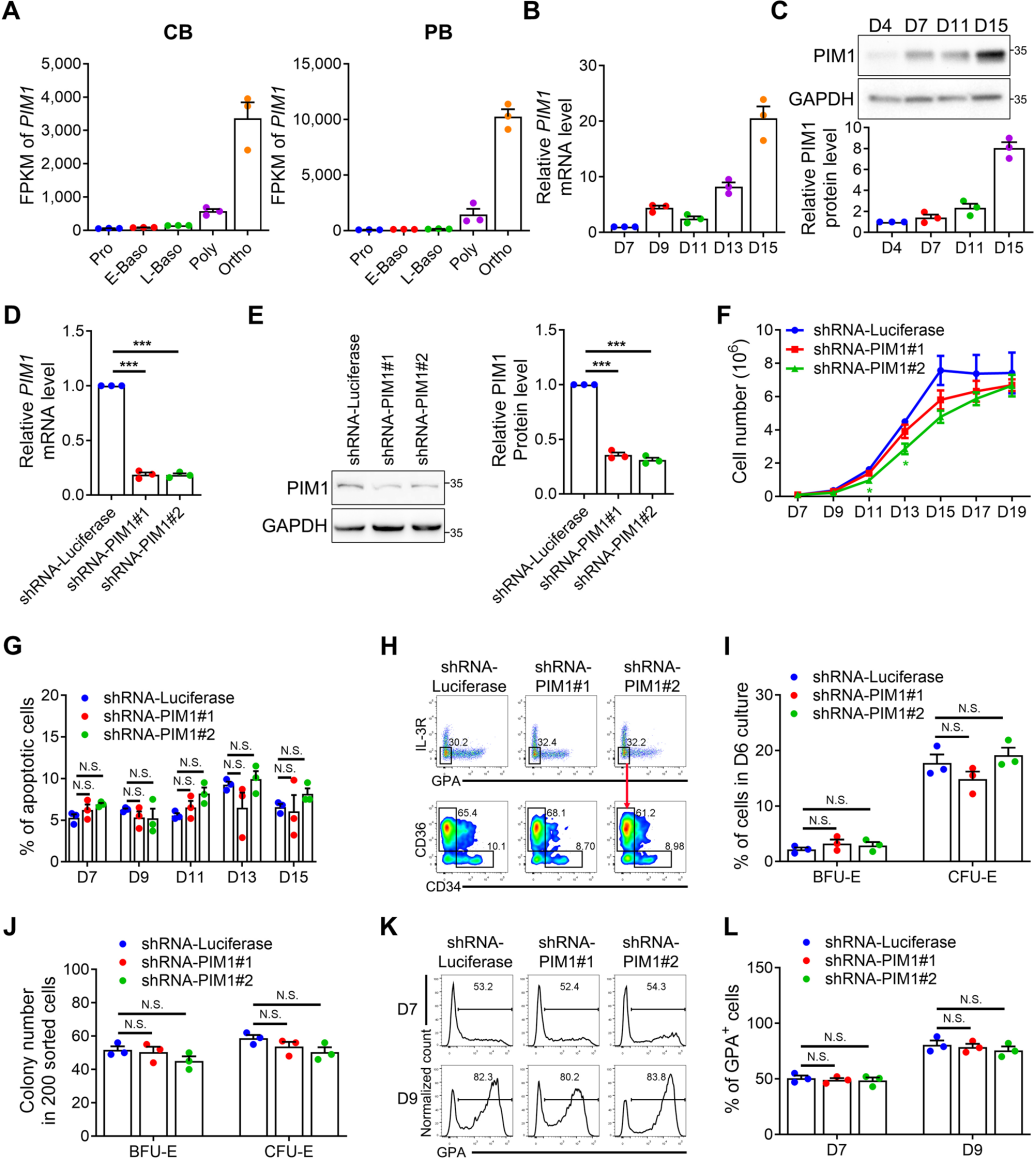
PIM1 did not affect the proliferation and differentiation of erythroid cells. **A** RNA-seq data revealing the gene expression levels of PIM1 at distinct stages of human terminal erythroid differentiation, including Pro, E-Baso, L-Baso, Poly, and Ortho. **B** qRT-PCR results showing *PIM1* mRNA expression levels in erythroblasts at indicated culture days. *β-Actin* was used as the internal reference. **C** Representative Western blotting analysis of PIM1 in erythroblasts at indicated culture days and its quantification. **D** qRT-PCR analysis showing the expression levels of *PIM1* in cultured erythroblasts transfected with shRNAs targeted to Luciferase (as control) or PIM1. *β-Actin* was used as the internal reference. **E** Representative Western blotting analysis showing PIM1 protein levels in shRNA-Luciferase or shRNA-PIM1 transfected erythroid cells. **F** Growth curve of cultured erythroid cells transfected with shRNA-Luciferase or shRNA-PIM1. **G** Quantitative analysis of apoptosis of cultured erythroid cells transfected with shRNA-Luciferase or shRNA-PIM1 at indicated culture days. **H** Representative flow cytometry profiles of early erythropoiesis detected by the expression of IL-3R, GPA, CD34 and CD36 at day 6 of culture. **I** Quantitative analyses of erythroid progenitors BFU-E and CFU-E at day 6 of culture. **J** Quantitative analysis showing colony forming ability of sorted luciferase or PIM1 knockdown erythroid progenitors BFU-E and CFU-E. **K**,** L** Representative flow cytometry analysis showing the percentage of GPA^+^ cells on day 7 and 9 of culture and its quantification. Data were presented as mean ± SEM. *P* values were determined by either One-way ANOVA (**D**,**E**,**G**,**I**,**J**,**L**) or Two-way ANOVA (**F**). Pro proerythroblasts, E-Baso early basophilic erythroblasts, L-Baso late basophilic erythroblasts, Poly polychromatic erythroblasts, Ortho orthochromatic erythroblasts, CB cord blood CD34^+^ cell derived erythroblasts, PB peripheral blood CD34^+^ cell derived erythroblasts, FPKM fragments per kilobase million, N.S. no statistic different. *P < 0.05, ***P < 0.001.

To investigate which protein kinases might play a regulatory role in erythroblast enucleation, we analyzed our previously published transcriptome data of Ortho derived from human cord blood CD34^+^ cells and found that PIM1 was the highest expressed protein kinase (Supplementary Table 1). Notably, PIM1 ranked among the top 25 highly expressed genes in Ortho^4^. Among the top 25 genes, the majority are hemoglobin-related genes, with PIM1 being the sole protein kinase (Supplementary Table 2). Next, we examined the expression profiling of PIM1. Data from the gene annotation portal BioGPS^26^ and human hematopoietic tissue gene expression database Bloodspot^27^ demonstrated that expression level of PIM1 was particularly high in the erythroid lineage (Supplementary Fig. 1A). Single-cell data of human bone marrow (BM)^28^ also showed that PIM1 expression gradually increased during erythroid development (Supplementary Fig. 1B). Additionally, our RNA-seq data of stage specific erythroblasts derived from human cord blood (CB) and peripheral blood (PB) CD34^+^ cells indicated that the expression of PIM1 in erythroid cells was up-regulated and highest in Ortho (Fig. 1A). The expression of PIM1 during human erythropoiesis was further confirmed by qRT-PCR (Fig. 1B) and Western blotting analysis (Fig. 1C). Furthermore, the conservative expression characteristic of Pim1 was also observed in mice. qRT-PCR and Western blotting analysis of different mouse tissues showed that Pim1 was highly expressed in mouse BM (Supplementary Fig. 1C, D). According to our RNA-seq data of mouse stage specific erythroblasts^4^, we found that, similar to the expression pattern in human erythroblasts, Pim1 was highly expressed in Ortho (Supplementary Fig. 1E). The tissue specific expression and unique expression pattern of PIM1 strongly suggest potentially important roles of PIM1 in erythropoiesis.

### Downregulation of PIM1 specifically impairs human erythroblast enucleation with no effects on proliferation and differentiation

To investigate the role of PIM1 in erythropoiesis, we first employed the shRNA-mediated knockdown approach during the in vitro erythroid differentiation of CD34^+^ cells obtained from human CB, an experimental system that is well suited for studying human erythropoiesis. ~70% knockdown efficiencies were achieved with two independent PIM1 shRNAs compared with the control Luciferase shRNA when examined by qRT-PCR (Fig. 1D) and Western blotting analysis (Fig. 1E). PIM1 has been reported to be an important signaling molecule that promotes cell proliferation and survival in multiple cancer cell types^29,30^. Surprisingly, in contrast to its roles in cancer cell lines, PIM1 knockdown exerted a minimal impact on cell growth during terminal erythroid differentiation (Fig. 1F), with shRNA-PIM1#2 treatment inducing a transient reduction in cell proliferation while shRNA-PIM1#1 showed no detectable effect. Annexin V staining also showed no statistical significance in apoptosis between PIM1 knockdown and control groups (Fig. 1G and Supplementary Fig. 2A). Notably, we observed a compensatory upregulation of PIM2 and PIM3 expression upon PIM1 knockdown (Supplementary Fig. 2B), suggesting functional redundancy among PIM kinase family members in maintaining proliferation and survival signals during erythroid differentiation. To rule out methodological differences and further confirm the role of PIM1 in cancer cells, we knocked down the expression of PIM1 using the same shRNAs in chronic myeloid leukemia cell line K562 and erythroleukemia cell line HEL. We observed inhibited cell growth and increased apoptosis in both cell lines (Supplementary Fig. 2C–F). These findings indicate that as an oncogene, PIM1 does not participate in the regulation of cell proliferation and apoptosis during normal erythropoiesis.

Erythropoiesis is a dynamic process containing early-stage erythropoiesis and terminal erythroid differentiation^2–4^. We first detected the effect of PIM1 knockdown on erythroid progenitors. Human erythroid progenitors BFU-E and CFU-E can be detected by the expression of specific cell surface markers and defined as IL-3^-^GPA^-^CD34^+^CD36^-^ cells and IL-3^-^GPA^-^CD34^-^CD36^+^ cells, respectively^31^. Flow cytometric results showed no difference in BFU-E or CFU-E populations following PIM1 knockdown (Fig. 1H, I and Supplementary Fig. 2G). Colony forming assay further confirmed that PIM1 knockdown did not affect the colony forming ability of BFU-E and CFU-E (Fig. 1J). Next, we checked the effect of PIM1 knockdown on terminal erythroid differentiation. The surface expression of GPA can be used to characterize the transition of early erythropoiesis to terminal erythroid differentiation^32,33^. Similar percentages of GPA^+^ cells were detected in the PIM1 knockdown and control groups monitored on day 7 and day 9 of culture (Fig. 1K, L). We further assessed terminal erythroid differentiation by the surface expression of protein Band3 and α4 integrin as previously reported^3^. No obvious influence on terminal erythroid differentiation was detected in the PIM1 knockdown groups. (Supplementary Fig. 2H, I). These findings indicate that PIM1 is not involved in differentiation of erythroid cells.

**Fig. 2:**
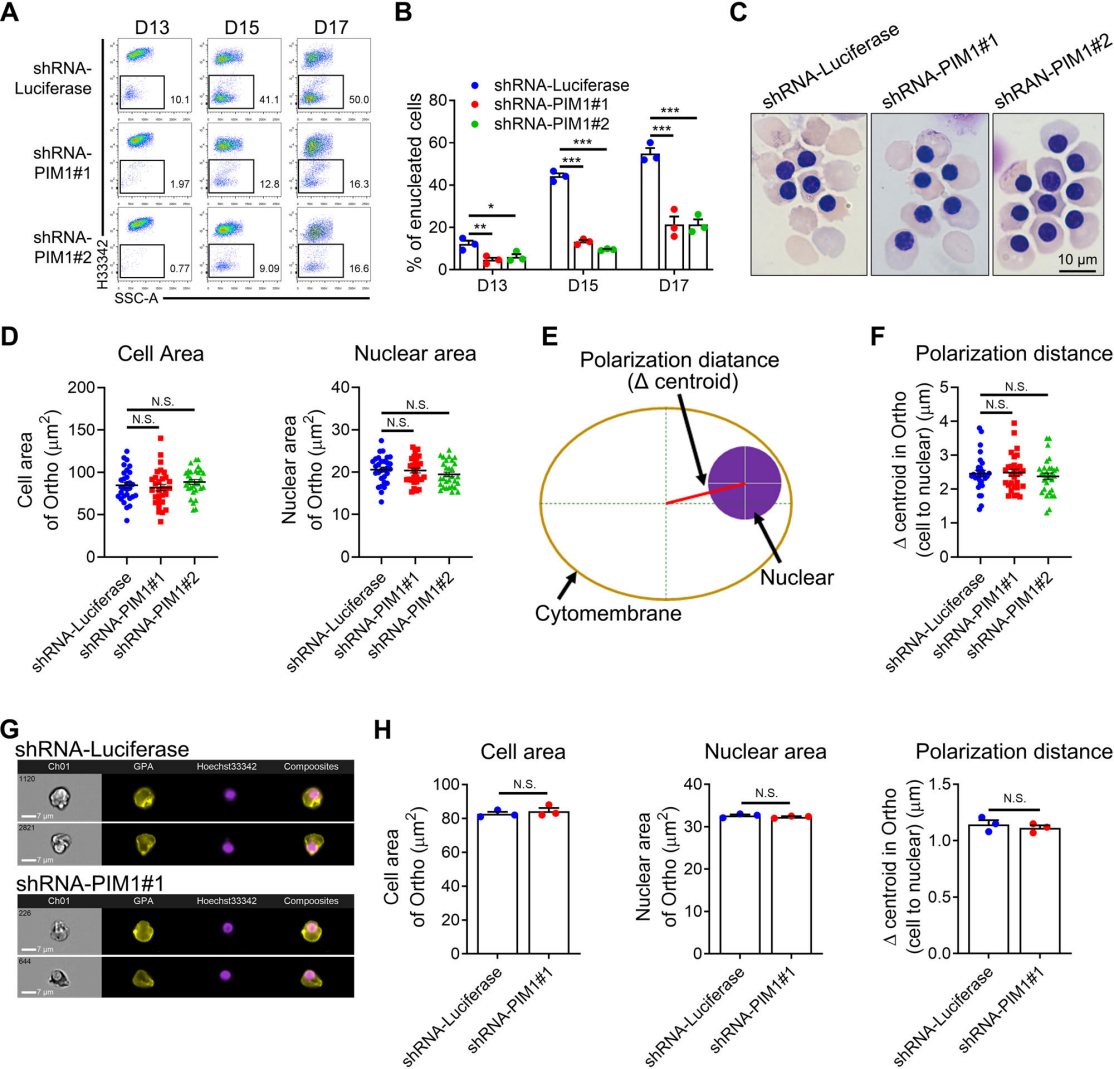
Loss of PIM1 impaired erythroblast enucleation without disturbing nuclear polarization. **A** Representative flow cytometry analysis showing the percentage of erythroblast enucleation in control and PIM1 knockdown groups. **B** Statistical analysis of the enucleation rate in each group from three independent experiments on days 13, 15 and 17. **C** Representative cytospin images of erythroid cells on day 17 of culture. Scale bar = 10 μm. **D** Statistical analysis of cell area (μm^2^), nuclear area (μm^2^) in shRNA-Luciferase or shRNA-PIM1 transfected Ortho according to cytospin images. *N* = 30. **E** Schematic diagram showing the pattern of nuclear polarization distance. **F** Statistical analysis of polarization distance (μm) in shRNA-Luciferase or shRNA-PIM1 transfected Ortho according to cytospin images. *N* = 30. **G** Representative imaging flow cytometry images of Ortho stained with GPA and Hoechst33342. **H** Quantitative analysis showing cell area (μm^2^), nuclear area (μm^2^) and polarization distance (μm) in shRNA-Luciferase or shRNA-PIM1 transfected Ortho detected by the imaging flow cytometry. Data were presented as mean ± SEM. *P* values were determined by either One-way ANOVA (**B**,** D**,** F**) or student’s *t* test (**H**). H33342 Hoechst33342, SSC side scatter, N.S. no statistic different. *P < 0.05, **P < 0.01, ***P < 0.001.

Erythroblast enucleation is the process by which Ortho expel their nucleus to generate enucleated reticulocytes, which can be detected by DNA staining such as Hoechst33342. We observed that PIM1 deficiency severely impaired the enucleation ability of erythroblasts, resulting in a 60% reduction compared to controls (Fig. 2A, B). The impaired enucleation was further confirmed by cytospin analysis (Fig. 2C). Consistent with these findings, treatment of day 13 erythroid cells with the PIM1 inhibitor SMI-4a for 48 h led to a dose-dependent decrease in enucleation (Supplementary Fig. 2J, K), reinforcing the critical role of PIM1 in erythroblast enucleation. Nuclear condensation and polarization are important prerequisite processes for erythroblast enucleation. To determine whether PIM1 affects nuclear condensation and polarization, we further analyzed the morphology of Ortho in cytospin. Our observations revealed no substantial alterations in cell or nuclear size, suggesting that PIM1 knockdown has no impact on nuclear condensation (Fig. 2D). Cell polarization was assessed by measuring the distance between the cytoplasmic and nuclear centers, referred to as the polarization distance (Fig. 2E). Notably, PIM1 knockdown did not exert any influence on polarization distance (Fig. 2F), indicating that PIM1 is not important for nuclear polarization during enucleation. To further substantiate this, we employed imaging flow cytometry (Fig. 2G and Supplementary Fig. 3). The results showed that PIM1 knockdown did not affect cell dimension, nuclear size, or polarization distance (Fig. 2H). Collectively, these findings demonstrate that PIM1 deficiency causes specific defects in human erythroblast enucleation.

### Conditional deletion of Pim1 in erythroid cells leads to generation of microcytic and hypochromic erythrocyte in vivo

**Fig. 3:**
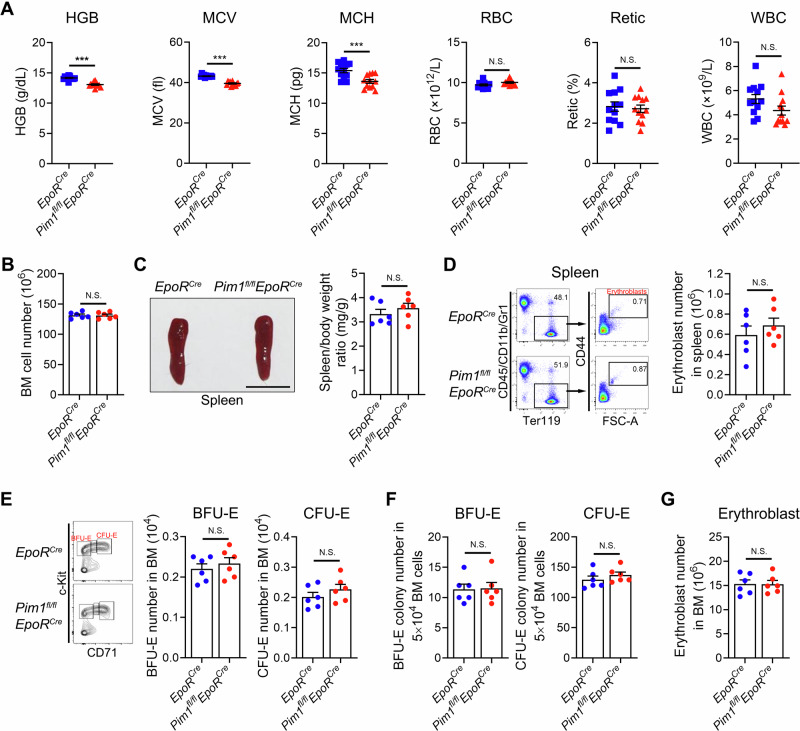
*Pim1*^*fl/fl*^*EpoR*^*Cre*^ mice exhibited microcytic and hypochromic red blood cells. **A** Peripheral blood parameters as indicated. *N* = 12. **B** BM cell number in 2 tibia and 2 femurs of *Pim1*^*fl/fl*^*EpoR*^*Cre*^ mice and controls. *N* = 6. **C** Comparison of the spleen morphology between the *Pim1*^*fl/fl*^*EpoR*^*Cre*^ mice and control group mice, along with statistical analysis of the spleen-to-body weight ratio. Scale bar = 1 cm. *N* = 6. **D** Flow cytometry analyses of terminal erythropoiesis and the quantification of erythroblast numbers in the spleen of *Pim1*^*fl/fl*^*EpoR*^*Cre*^ mice and control mice. *N* = 6. **E** Flow cytometry analyses of early erythropoiesis and the quantification of BFU-E and CFU-E numbers in the BM of *Pim1*^*fl/fl*^*EpoR*^*Cre*^ mice and control group mice. *N* = 6. **F** Numbers of BFU-E colonies and CFU-E colonies detected by colony forming assay in the BM of *Pim1*^*fl/fl*^*EpoR*^*Cre*^ mice and controls. *N* = 6. **G** Quantification of erythroblasts numbers in the BM of *Pim1*^*fl/fl*^*EpoR*^*Cre*^ mice and control mice. *N* = 6. Data were presented as mean ± SEM. *P* values were determined by student’s *t* test. HGB hemoglobin content, MCV mean corpuscular volume, MCH mean corpuscular hemoglobin, RBC red blood cell count, Retic reticulocyte count, WBC white blood cell count, BM bone marrow, FSC forward scatte, N.S. no statistic different. ***P < 0.001.

Whole-body PIM1 knockout mice exhibited no significant phenotypic differences except for the production of microcytic erythroid cells^34^. To exclude potential confounding effects from other cell types, we generated *Pim1*^*fl/fl*^*EpoR*^*Cre*^ mice in which Pim1 will be deleted in EpoR expressing cells including erythroid cells and erythroblastic island (EBI) macrophages^35^ (Supplementary Fig. 4A). Supplementary Fig. 4B shows representative genotyping results. The deletion of Pim1 in Ter119^+^ erythroid cells was confirmed by Western blotting analysis (Supplementary Fig. 4C, D). *Pim1*^*fl/fl*^*EpoR*^*Cre*^ mice were viable and fertile, with no obvious gross abnormalities. Compared with control mice, *Pim1*^*fl/fl*^*EpoR*^*Cre*^ mice had no significant hematologic abnormalities except for decreases in hemoglobin content (HGB), mean corpuscular volume (MCV) and mean corpuscular hemoglobin (MCH) (Fig. 3A). The unchanged number of BM cells, spleen size, and number of erythroblasts in the spleen indicate that there was no compensatory erythropoiesis in the mice (Fig. 3B–D).

Next, we investigated whether ablation of Pim1 leads to the generation of abnormal erythrocytes by affecting erythroid differentiation or survival. Mouse BM erythroid progenitors can be identified using flow cytometry based on specific surface markers, as we previously reported^35^. BFU-E and CFU-E are identified as CD71^low/-^ cells and CD71^high^ cells within the Lin^-^CD16/32^-^CD41^-^CD34^-^Sca1^-^c-Kit^+^ cell population, respectively^35^. The number of erythroid progenitors was not changed in *Pim1*^*fl/fl*^*EpoR*^*Cre*^ mice compared to controls (Fig. 3E and Supplementary Fig. 4E). Colony forming assay showed that there was no remarkable difference in the colony number of erythroid progenitors in mouse BM (Fig. 3F), further validating that Pim1 is not essential for early erythropoiesis in mice. Terminal erythroid differentiation in mice was assessed using surface markers, including Ter119 and CD44, as previously reported^36^. The gating strategy of terminal erythropoiesis was shown in Supplementary Fig. 4E. Conditional Pim1 deficiency in mice had no effect on terminal erythroid differentiation, as evidenced by the unchanged proportion of erythroblasts at each stage (Supplementary Fig. 4F) and the number of erythroblasts (Fig. 3G) compared to controls. No differences in EBI macrophages were detected between control and *Pim1*^*fl/fl*^*EpoR*^*Cre*^ mice (Supplementary Fig. 4G), despite expression of *Pim1* in these cells^37^. Consistent with human erythropoiesis, apoptosis was not changed in erythroblasts of *Pim1*^*fl/fl*^*EpoR*^*Cre*^ mice (Supplementary Fig. 4H). Together, our results indicate that deletion of Pim1 in erythroid cells leads to generation of microcytic and hypochromic red cells without affecting differentiation and survival of erythroid cells in mice.

### Erythroid cells lacking Pim1 exhibit abnormal enucleation in mice

**Fig. 4:**
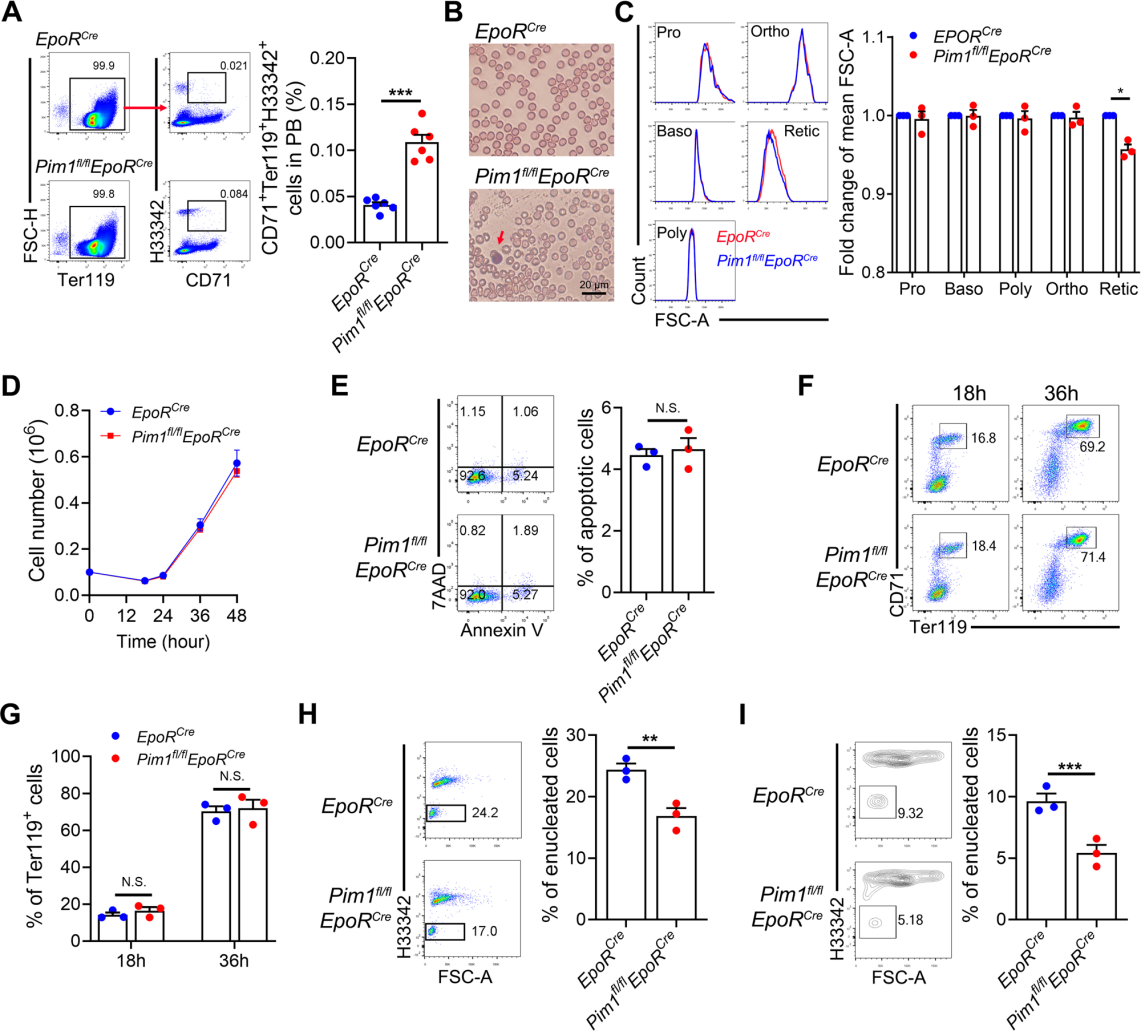
Erythroblast enucleation was impaired in *Pim1*^*fl/fl*^*EpoR*^*Cre*^ mice. **A** Representative flow cytometry analysis and quantitative results of the percentage of CD71^+^Ter119^+^Hoechst33342^+^ cells in peripheral blood of *Pim1*^*fl/fl*^*EpoR*^*Cre*^ mice and controls. *N* = 6. **B** Representative cytospin images showing the peripheral blood cells. The red arrow indicating nucleated erythroblasts. **C** Flow cytometry analyses and statistical results showing the mean of FSC-A, which indicates cell size, of terminal differentiated erythroid cells in *Pim1*^*fl/fl*^*EpoR*^*Cre*^ mice and control mice. **D** Growth curves of erythroid cells derived from BM lineage^-^ cells of *Pim1*^*fl/fl*^*EpoR*^*Cre*^ mice and control mice. **E** Flow cytometry analyses and quantitative results showing apoptotic rate of cultured erythroid cells of *Pim1*^*fl/fl*^*EpoR*^*Cre*^ mice and control mice at 18 hours (h). **F**,** G** Flow cytometry analyses and quantitative results showing Ter119^+^CD71^+^ cell percentage of cultured erythroid cells of *Pim1*^*fl/fl*^*EpoR*^*Cre*^ mice and control mice at 18 h and 36 h. **H** Flow cytometry analyses and quantitative results showing the percentage of enucleated erythroblasts of cultured erythroid cells of *Pim1*^*fl/fl*^*EpoR*^*Cre*^ mice and control mice at 48 h. **I** Flow cytometry analyses showing the enucleation rate of sorted BM Poly form *Pim1*^*fl/fl*^*EpoR*^*Cre*^ mice and control mice cultured for 18 h and their quantification. Data were presented as mean ± SEM. *P* values were determined by student’s *t* test. FSC forward scatter, H33342 Hoechst33342, N.S. no statistic different. *P < 0.05, **P < 0.01, ***P < 0.001.

Given that knockdown of PIM1 impaired human erythroblast enucleation, we next examined whether deletion of Pim1 affects enucleation in mice. Interestingly, the proportion of Ter119^+^CD71^+^Hoechst33342^+^ nucleated erythroblasts in peripheral blood, which reflects impaired erythropoiesis^9^, was significantly higher in *Pim1*^*fl/fl*^*EpoR*^*Cre*^ mice than that in control mice (Fig. 4A). Cytospin analysis confirmed the presence of nucleated erythroblasts in the peripheral blood of *Pim1*^*fl/fl*^*EpoR*^*Cre*^ mice (Fig. 4B). Furthermore, although cell size of erythroblasts at each stage did not change in BM of *Pim1*^*fl/fl*^*EpoR*^*Cre*^ mice, the size of reticulocytes decreased significantly (Fig. 4C). The reduction of cell size occurring at the stage between Ortho to reticulocytes suggest alterations in erythroblast enucleation in *Pim1*^*fl/fl*^*EpoR*^*Cre*^ mice.

An in vitro differentiation system, which involves key stages of terminal erythroid differentiation, is commonly used to evaluate mouse terminal erythropoiesis and enucleation^17^. Hematopoietic stem and progenitor cells (HSPC, lineage^-^ cells) were isolated from mouse BM by depletion of lineage^+^ cells and differentiated in vitro for 48 h. We found that although Pim1 deficiency had no significant effect on cell proliferation (Fig. 4D), apoptosis (Fig. 4E) and differentiation (Fig. 4F, G and Supplementary Fig. 4I), the proportion of erythroblast enucleation was significantly reduced compared to the control groups (Fig. 4H). To further confirm the role of Pim1 on enucleation, we sorted out Poly from mouse BM and cultured for 18 h to let them enucleate. Similar to previous results, the enucleation rate of sorted erythroid cells was significantly lower than that of control cells (Fig. 4I). Dysregulation of cell cycle exit represents a critical contributor to enucleation defects. Flowcytometry analyses revealed comparable Ki67 expression levels in Ortho from *Pim1*^*fl/fl*^*EpoR*^*Cre*^ mice relative to controls, indicating that Pim1 ablation does not interfere with cell cycle exit processes (Supplementary Fig. 4J). Taken together, these data indicate that PIM1 has a conserved function on regulating erythroblast enucleation without controlling cell proliferation, differentiation, and survival during normal terminal erythropoiesis.

### PIM1 deficiency decreases mitochondrial mass in Ortho

**Fig. 5:**
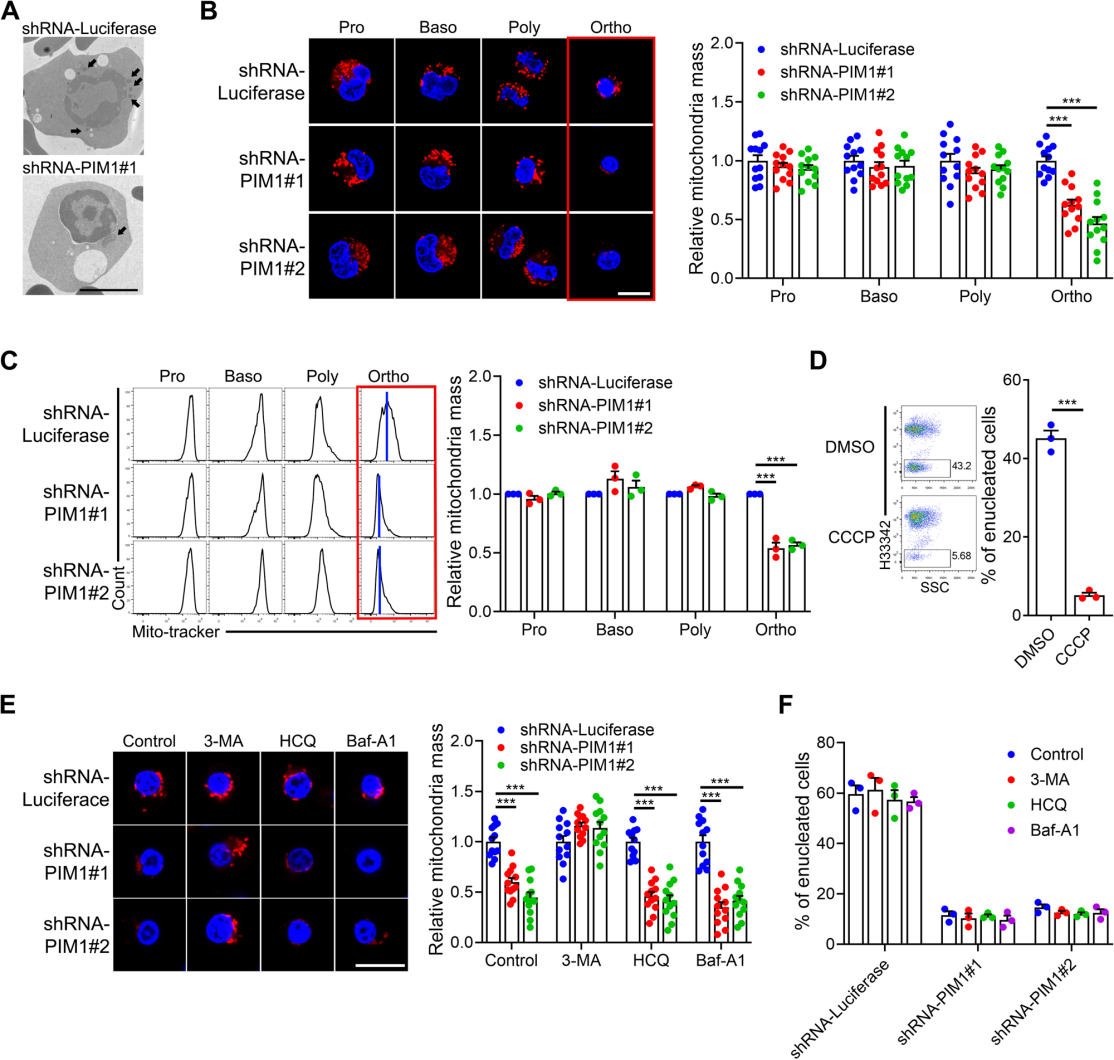
Mitochondrial mass decreased in PIM1 knockdown Ortho. **A** Representative transmission electron microscopy images showing the decrease of mitochondria number in PIM1 knockdown Ortho. Scale bar = 5 μm. **B** Fluorescent images showing the mitochondria mass in indicated human cultured erythroid cells transfected with shRNA-Luciferase or shRNA-PIM1 and the quantification of mitochondria mass in indicated cells. Mitochondria was labeled with Mito-tracker red and cell nucleus were labeled with Hoechst33342. Scale bar = 10 μm. *N* = 12. **C** Flow cytometry analyses and statistical results showing the mitochondria mass in indicated human cultured erythroid cells transfected with shRNA-Luciferase or shRNA-PIM1. The blue lines indicated the medium of fluorescent intensity. **D** Flow cytometry analyses and statistical results showing the enucleation rate of cultured day 13 erythroid cells treatment with mitochondrial function inhibitor CCCP for 2 days. **E** Fluorescent images and statistical results showing the mitochondria mass in Ortho transfected with shRNA-Luciferase or shRNA-PIM1 after treatment with autophagy inhibitor 3-MA, HCQ or Baf-A1 for 2 days. Mitochondria was labeled with Mito-tracker red and cell nucleus were labeled with Hoechst33342. Scale bar = 10 μm. *N* = 12. **F** Statistical analyses showing the enucleation rate of cultured day 15 erythroid cells transfected with shRNA-Luciferase or shRNA-PIM1 treated with autophagy inhibitor 3-MA, HCQ or Baf-A1 for 2 days. Data were presented as mean ± SEM. *P* values were determined by either One-way ANOVA (**B**,** C**,** E**,** F**) or student’s *t* test (**D**). SSC side scatter, DMSO dimethyl sulfoxide, CCCP carbonyl cyanide m-chlorophenyl hydrazine, 3-MA 3-methyladenine, HCQ hydroxychloroquine, Baf-A1 bafilomycin A1, H33342 Hoechst33342. ***P < 0.001.

To investigate the mechanisms by which PIM1 regulates enucleation, we carried out transmission electron microscopy analysis. The results showed a remarkable reduction in the number of mitochondria in PIM1-knockdown Ortho (Fig. 5A). Furthermore, both confocal microscopy analysis and flow cytometric analysis revealed that mitochondrial mass, as indicated by Mito-tracker, was significantly decreased in PIM1-deficient Ortho, but not in Pro, Baso and Poly (Fig. 5B, C), suggesting that defective mitochondria function due to decreased mitochondria mass may contribute to the impaired enucleation of the PIM1-deficient erythroblasts. This assumption is supported by our finding that disruption of mitochondrial function by the oxidative phosphorylation uncoupler carbonyl cyanide 3 chlorophenylhydrazone (CCCP) potently inhibited erythroblast enucleation (Fig. 5D).

The clearance of organelles through autophagy is a crucial process during the terminal differentiation of erythroid cells^38^. To investigate the cause of the decreased mitochondrial mass in PIM1-deficient Ortho, we assessed the level of autophagy using LC3B as a marker. We observed that autophagy levels were significantly elevated in PIM1 deficient Ortho, whereas changes were minimal in Pro (Supplementary Fig. 5), suggesting a relationship between increased autophagy and decreased mitochondria mass in PIM1 knockdown Ortho. We then examined whether inhibition of autophagy can rescue mitochondria mass. We found that only autophagy initiation inhibitor 3-Methyladenine (3-MA), but not late-stage autophagy inhibitors Hydroxychloroquine (HCQ), and Bafilomycin A1 (Baf-A1), restored mitochondrial mass (Fig. 5E). However, even though 3-MA rescued the mitochondria mass, it did not rescue enucleation of PIM1-knockdown erythroblasts (Fig. 5F), strongly suggesting that in addition to autophagy-mediated mitochondrial mass, other PIM1 deficiency-driven changes may contribute significantly to the impaired enucleation.

### PIM1 knockdown leads to decreased phosphorylation of GTPase-related proteins in Ortho

**Fig. 6:**
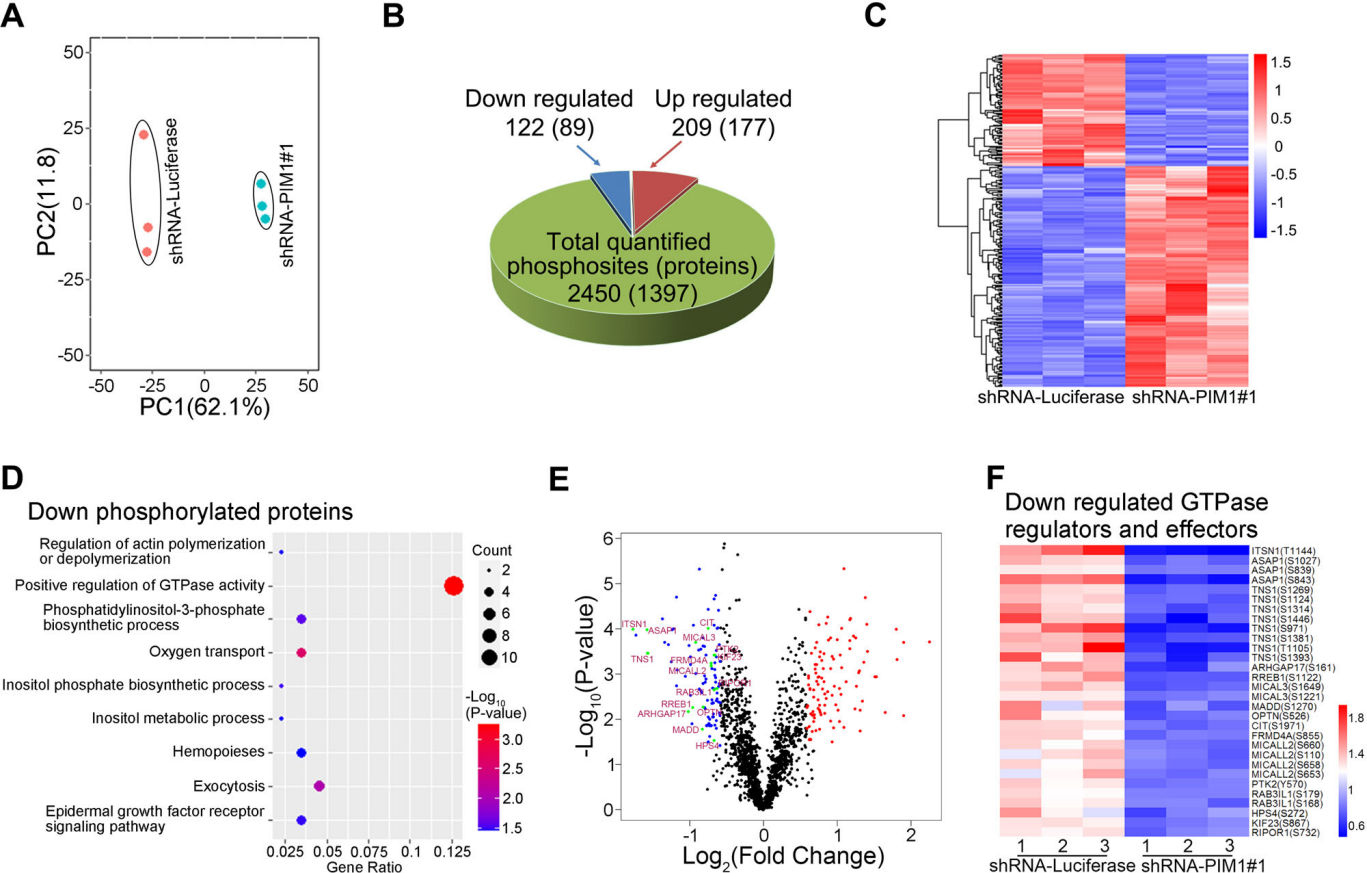
PIM1 controlled erythroblast enucleation by regulating the activity of GTPase-related proteins. **A** Principal components analysis of Ortho transfected with shRNA-PIM1#1 versus control cells. **B** Pie charts illustrating the numbers of altered and unchanged phosphosites and proteins (the numbers in parentheses) following PIM1 knockdown in Ortho. **C** Heatmap displaying differential phosphosites between control and shRNA-PIM1#1 treated samples. **D** Bubble chart of enriched GO terms of down phosphorylated proteins in Ortho transfected with shRNA-PIM1 versus control cells. **E** Volcano plot of differentially phosphorylated proteins, with downregulated GTPases regulators and effectors labeled out. **F** Heatmap displaying the downregulated phosphosites of GTPase-related proteins in PIM1 deficient erythroid cells. PC principal component.

To further explore the potential mechanisms by which PIM1 regulates erythroblast enucleation, we performed quantitative phosphoproteomics analysis on human Ortho. In total, 2450 phosphosites (localization probability >0.75) located on 1397 proteins were quantified (Supplementary Data 1). Principal component analysis (PCA) of 3 biological replicates of PIM1 knockdown and control group revealed clear segregation between the groups, suggesting reproducible quantification (Fig. 6A). Differentially phosphorylated proteins were defined as those containing at least 1 phosphosite with *P* < 0.05 and fold change >1.5. Phosphorylation levels of 209 phosphosites (177 proteins) were upregulated, while 122 phosphosites (89 proteins) were downregulated (Fig. 6B). The heatmap of the differentially phosphorylated proteins was shown in Fig. 6C. The differentially phosphorylated phosphosites are listed in Supplementary Data 2. In line with prior research, our analysis revealed a significant elevation in the phosphorylation levels of key proteins implicated in mitochondrial quality control and autophagy, including BNIP3L, SQSTM1, and WDFY3 in PIM1-knockdown Ortho (Supplementary Fig. 6). Given that PIM1 is a protein kinase, our subsequent analyses focused on down-regulated proteins to explore downstream pathways of its direct regulation. Gene ontology (GO) analysis revealed that the term of positive regulation of GTPase activity exhibited the most significant differences and encompassed the largest number of genes (Fig. 6D). All the down phosphorylated proteins and phosphosites related to GTPase pathways, including GTPase regulators and effectors, were labeled in the volcano plot (Fig. 6E) and the heatmap (Fig. 6F), respectively. Their functional annotation and expression during erythropoiesis are shown in Supplementary Table 3 and Supplementary Fig. 7, respectively. Further GO analysis revealed that these PIM1-regulated GTPase related phosphoproteins were functionally clustered in critical pathways governing vesicle trafficking, including the pathways of exocytosis (ITSN1, MICAL3, RAB3IL1) and Golgi to plasma membrane protein transport (ASAP1, OPTN), and cytoskeletal reorganization, including GO terms of positive regulation of epithelial cell migration (RREB1, PTK2) and regulation of actin cytoskeleton organization (ARHGAP17, CIT) (Supplementary Table 4). It is well known that the assembly of F-actin^16–20^ and vesical trafficking, especially endocytosis^22,23^, are essential for erythroblast enucleation. Therefore, these findings underscore the critical upstream regulatory role of PIM1 in erythroblast enucleation by modulating GTPase activity.

### PIM1 knockdown affects F-actin organization and vesicle transport in Ortho

**Fig. 7:**
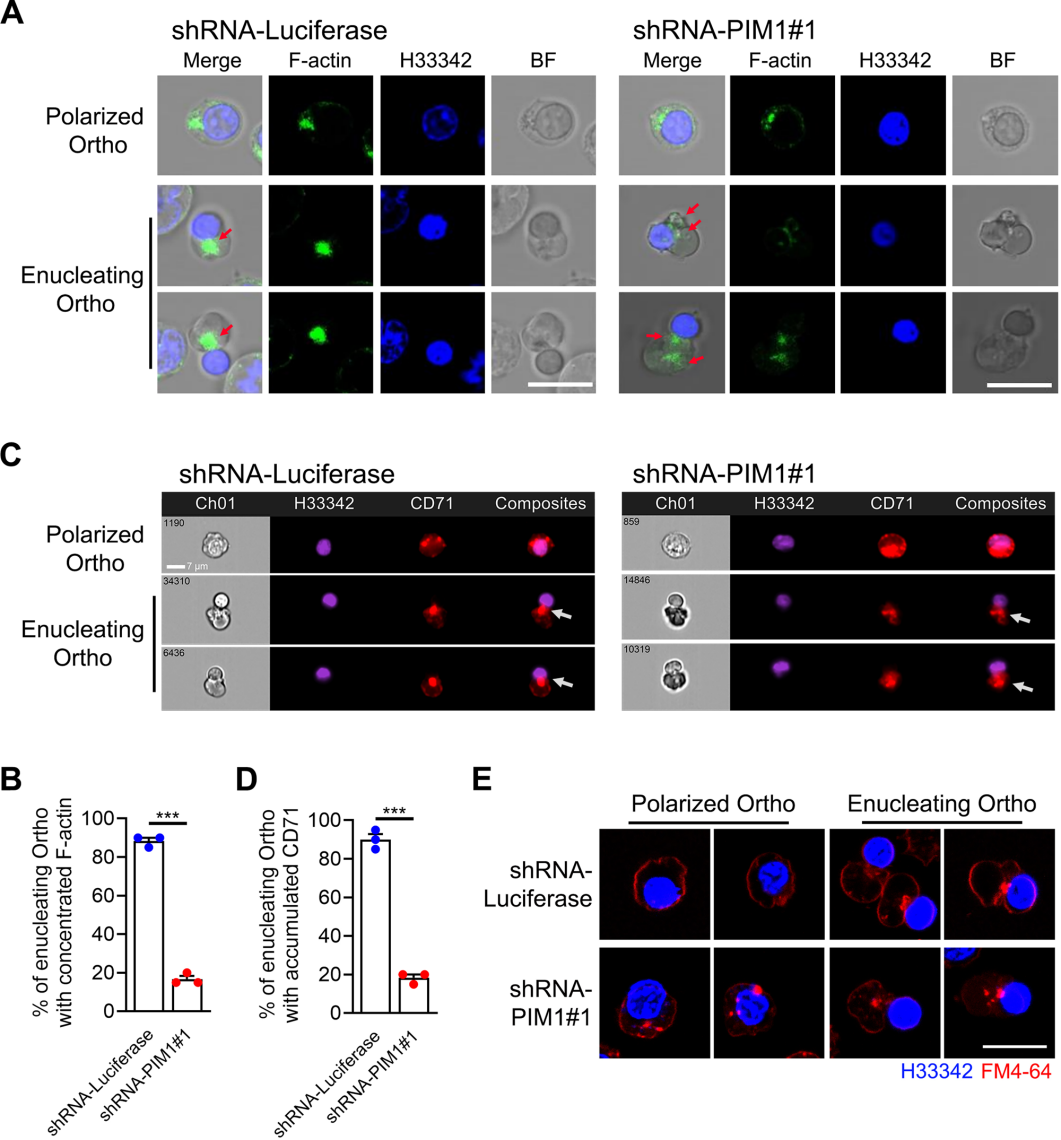
PIM1 controlled F-actin network and vesicle trafficking in Ortho. **A** Fluorescence images showing the localization of F-actin in polarized and enucleating Ortho transfected with shRNA-Luciferase or shRNA-PIM1. Scale bar = 10 μm. **B** Quantitative analysis showing the percentage of enucleating Ortho with concentrated F-actin between nucleus and cytoplasm in PIM1 knockdown groups and controls. **C** Representative imaging flowcytometry images showing the internalization of CD71 in polarized and enucleating Ortho transfected with shRNA-Luciferase or shRNA-PIM1. **D** Quantitative analysis showing the percentage of enucleating Ortho with accumulated CD71 between nucleus and cytoplasm in PIM1 knockdown groups and controls. **E** Fluorescence images depicting the internalization of membrane-impermeable dye FM4-64 in polarized and enucleating Ortho with or without PIM1 deficiency. Scale bar = 10 μm. Data were presented as mean ± SEM. *P *values were determined by student’s *t* test. H33342 Hoechst33342, BF bright field. ***P < 0.001.

We next investigated whether changes in the activity of GTPase regulators and effectors resulting from PIM1 knockdown cause disruption of F-actin network organization and endocytosis. F-actin was labeled and tracked using phalloidin. We found that although the distribution of F-actin did not show significant differences in polarized Ortho, it varied markedly in enucleating Ortho: F-actin in control cells was concentrated at the interface between nucleus and cytoplasm, whereas in PIM1-knockdown cells, F-actin was dispersed and exhibited reduced fluorescence intensity (Fig. 7A, B), indicating the regulatory role of PIM1 in F-actin organization. Similar results were observed in mouse Ortho (Supplementary Fig. 8A).

Then, endocytosis was visualized using membrane-impermeable fluorescent reagents. We employed imaging flow cytometry analysis to examine endocytosis in Ortho through CD71 fluorescent staining^39^. The results revealed that after PIM1 knockdown, the internalized membrane could not accumulate between the cytoplasm and nucleus (Fig. 7C, D). We then used a membrane-impermeable red fluorescent dye FM4-64 to confirm the regulatory role of PIM1 on endocytosis in Ortho. The results showed that the knockdown of PIM1 led to the premature aggregation of endocytic vesicles in polarized Ortho, which was contradictory to the observation that vesicles accumulated occurred at late stage of nuclear extrusion^22,23^. Consistently, these internalized vesicles failed to converge effectively in the region between cytoplasm and nucleus in enucleating Ortho (Fig. 7E). Dispersed endocytic vesicles were also observed in mouse Ortho (Supplementary Fig. 8B). In addition, CCCP treatment also resulted in inhibited accumulation of F-actin and vesicles between the nucleus and cytoplasm in enucleating Ortho, showing similar effects to those observed following PIM1 knockdown (Supplementary Fig. 8C, D). In summary, our data demonstrate the central regulatory function of PIM1 in ensuring the normal progression of erythroblast enucleation, through regulation of cytoskeletal dynamics and vesicular transport homeostasis.

## Discussion

Erythroblast enucleation is a unique process during terminal erythropoiesis in mammals^6^. Numerous factors, including epigenetic factors^10–13^, cytoskeletal proteins^14–22^, Rho GTPases^16,17^, and transcription factors^40–43^, are involved in the regulation of erythroblast enucleation. However, the upstream regulators controlling erythroblast enucleation remain unclear. Given the critical roles of protein kinases in regulating cellular processes, we herein report the significant involvement of PIM1, the protein kinase with the highest expression in Ortho, in modulating mammalian erythroblast enucleation. Unlike other protein kinases such as CRIK^41^, CDK9^44^, p38α^45^ and AURKA^14^, the absence of PIM1 has minimal impact on the differentiation of erythroid cells but specifically affects the enucleation process, highlighting the distinct function of this kinase. Through a combination of in vitro and in vivo experiments, we have elucidated the critical role of PIM1 in modulating actin polymerization and maintaining vesicle trafficking homeostasis via the GTPase-associated pathway in Ortho, thereby impacting mammalian erythroblast enucleation.

PIM1 was first identified in lymphocytic carcinoma^46^ and is well documented for its role in regulating cell proliferation and survival^30^. However, our study of normal erythroid cells revealed a different outcome: the deletion of PIM1 does not affect cell proliferation or apoptosis. This discrepancy can be explained by two main factors. First, the cellular context is crucial. Cancer cells, which require high anti-apoptotic activity to support their abnormal growth, depend heavily on PIM1 for survival and proliferation. In contrast, erythroid cells operate in a distinct physiological environment where homeostatic mechanisms are finely tuned, possibly making them less reliant on PIM1 for anti-apoptotic functions. Second, the compensatory capabilities of the PIM kinase family members, PIM2 and PIM3, should not be overlooked. And our data showed increased expression of both PIM2 and PIM3 in PIM1 knockdown erythroblasts compared to controls. These kinases share similar substrates and activate analogous signaling pathways^47^. This functional redundancy suggests that in the absence of PIM1, PIM2 and/or PIM3 may intervene to sustain critical signaling routes, masking the loss of PIM1 in erythroid cells. Nonetheless, the precise molecular mechanisms underlying this compensation and the variation in compensatory efficacy across different cellular contexts remain largely elusive. Further research is necessary to delve into the mechanisms underlying the diverse roles of PIM1 in both physiological and pathological contexts.

Our findings reveal that PIM1 regulates mitochondrial mass in Ortho. Mitochondria play a crucial role in erythroblast enucleation, as evidenced by the marked reduction in enucleation rates following treatment with the mitochondrial uncoupling agent CCCP and the specific localization during erythroblast enucleation^48^. Previous studies have shown that PIM1 regulates mitochondrial mass by inhibiting mitochondrial fragmentation and apoptosis in cancer cells^49,50^. Nevertheless, in Ortho, modulation of mitochondrial mass by PIM1 appears to involve autophagy, the pathway responsible for organelle clearance during terminal erythropoiesis^38^. Inhibition of autophagy initiation can mitigate the reduction in mitochondrial mass induced by PIM1 knockdown. Surprisingly, even when mitochondrial mass is restored to normal levels, PIM1-deficient cells continue to exhibit enucleation defects. This suggests that the regulatory role of PIM1 in enucleation extends beyond its control over mitochondrial function, potentially attributed to its broad variety of downstream substrates. Further exploration has revealed PIM1’s involvement in modulating actin polymerization and vesicle trafficking pathways. These findings illustrate the important role of PIM1 in coordinating multiple pathways to regulate erythroblast enucleation.

GTPases, which play key roles in regulating complex cellular processes and subcellular events such as cell differentiation, proliferation, and vesicle trafficking, are critical for erythroblast enucleation. For instance, downregulation of Rab10 and its downstream activator EHBP1L1 impaired erythroblast enucleation by disrupting nuclear polarization^39^. Rho GTPases and their downstream effector mDia2 are essential for enucleation by their regulation roles on the formation of the contractile actin ring^16,17^. Additionally, Cdc42, a member of the Ras superfamily, regulates erythroid polarization and the formation of contractile actomyosin rings during enucleation^51^. While it is well established that GTPases regulate erythroblast enucleation by modulating functional processes such as the actin cytoskeleton dynamics and vesicular transport, their upstream regulatory mechanisms remain obscure. Our study demonstrates that PIM1 plays a pivotal role in regulating GTPase-related pathways. Knockdown of PIM1 in Ortho results in reduced phosphorylation of several GTPase-associated proteins. In terms of mechanism, PIM1 can affect erythroblast enucleation by regulating the aggregation of vesicles and F-actin in Ortho. Previous reports have indicated that disruption of vesicle trafficking leads to enucleation defects^22^, while cells undergoing normal enucleation accumulate vesicles between the nucleus and cytoplasm to facilitate this process^52^. On the other hand, the assembled F-actin between cytoplasm and nucleus during enucleation helps achieve complete separation of the reticulocyte from the nucleus^16,20^. These findings elucidate the regulatory role of PIM1 in orchestrating GTPase-related pathways to regulate F-actin assembly and endocytosis in Ortho, thereby enhancing our comprehension of the inherent mechanisms that govern enucleation. It is worth noting that although our study revealed a significant impact of PIM1 knockdown on the phosphorylation levels of GTPase-related proteins, we did not observe notable changes in the phosphorylation of previously reported enucleation-regulating GTPase-associated proteins or other key regulators such as KLF1. This finding suggests that PIM1’s regulatory role in erythroblast enucleation operates through a relatively independent pathway, coordinating with other factors to maintain the orderly progression of this process.

We propose that disordered erythroblast enucleation contributed to the observed decrease in MCV in vivo. It has long been postulated that there is a positive correlation between MCV and MCH^53,54^. Consequently, factors affecting hemoglobin synthesis, including iron deficiency, restricted iron utilization, and abnormal globin, have been regarded as pivotal determinants of MCV magnitude. Nevertheless, a paradoxical increase in MCV accompanied by a marked decrease in MCH was observed in mice following erythropoietin administration^55^, suggesting that the modulation of MCV involves mechanisms beyond hemoglobin synthesis. Our results show that erythroblast size remains unchanged before enucleation, including Pro, Baso, Poly and Ortho stages, but undergoes a significant reduction thereafter. We hypothesize that PIM1 deficiency impairs enucleation, leading to the expulsion of not only the nucleus but also excess cytoplasm, thereby reducing cell size. On another note, PIM1-deficient mice exhibit a phenotype of microcytic hypochromic red blood cells, implicating PIM1’s involvement in the regulation of hemoglobin synthesis. The reduced mass of mitochondria, a critical site for heme synthesis, regulated by PIM1, may be a significant factor contributing to the decreased MCH in erythroid cells lacking PIM1. Nonetheless, whether hemoglobin synthesis regulated by PIM1 plays a role in enucleation remains ambiguous and warrants further investigation.

In summary, our study identifies a novel role of PIM1 in erythroblast enucleation. PIM1 regulates nuclear extrusion by modulating GTPase-related pathways that control actin filament organization and vesical trafficking during erythroblast enucleation both in vitro and in vivo, suggesting conserved mechanisms across mammals. Our findings significantly advance our understanding of the regulatory mechanisms underlying erythroblast enucleation.

## Materials and methods

### Mice, antibodies, and primers

EpoR-tdTomato-Cre (*EpoR*^*Cre*^) mice^35^ were described previously. Pim1-floxed (*Pim1*^*fl/fl*^) mice were generated using the CRISPR/Cas9 technology at Biocytogen. In detail, based on the mouse *Pim1* gene structure, two loxP sites were selected for insertion into intron 4 and a non-conserved region downstream of the 3’ untranslated region (UTR), respectively. The 1500 bp 5’ homologous arm, the loxP-flanked *Pim1* genomic sequence, and the 1500 bp 3’ homologous arm were amplified. These PCR products were then cloned into a targeting vector. To identify high activity sgRNAs, several sgRNAs were designed and the activity of sgRNA was validated by using a UCA kit (Beijing Biocytogen Co., Ltd, China). The sgRNAs GTCTTTGGGCCGATGTGTAA (targeting intron 4) and AAGGGCATGTATAGTTCCAC (targeting downstream of 3’ UTR) were chosen and constructed into pT7-sgRNA plasmid (pT7-gRNA was a gift from Wenbiao Chen. Addgene plasmid # 46759. http://n2t.net/addgene:46759; RRID: Addgene_46759; PubMed 23918387). Then sgRNAs were transcribed in vitro. To generate genome edited mice, Cas9 protein, sgRNAs and the targeting vector were co-injected into pro-nuclei of C57Bl/6 N one cell stage zygotes. Genetically targeted mice were screened by polymerase chain reaction (PCR) using two different pairs of primers: EGE-ZYY-019-L-GT-F: TGGAGAAGGACCGGATTTCCGATTG, cKO-3’-DO-R: GACGCCTAGATTGTGCTACTCTCAGCT; cKO-5’-DO-F: GACGCCTAGATTGTGCTACTCTCAGCT, EGE-ZYY-019-R-GT-R: GACGCCTAGATTGTGCTACTCTCAGCT. The first primer pair amplified a 5564 bp band and the second a 5486 bp band from *Pim1* loxP allele. Southern blotting was used for further confirm correct recombination. BclI and NdeI were chosen as restriction enzyme cutting sites. 5’ Probe-B confirmed correct recombination while A Probe-A checked for random insertion. Animal protocols were reviewed and approved by the Animal Ethics Committee of the Institute of Zhengzhou University. *EpoR*^*Cre*^ mice served as the control group, while *Pim1*^*fl/fl*^*EpoR*^*Cre*^ mice were used as the experimental group. Maintained within specific pathogen-free (SPF) barrier facilities at Zhengzhou University, the mice resided under meticulously controlled environmental conditions encompassing stable temperature and humidity levels. They were subjected to a regular 12-h light/dark cycle and granted unrestricted access to both water and standard laboratory diet. Littermates were assigned to each group based on genotyping results. Both female and male mice (3–5 months old) were used with no observed sex differences. Euthanasia was performed via cervical dislocation under isoflurane anesthesia prior to downstream procedures. Sample sizes were established by the investigators drawing upon prior experimental experience; specific numbers for each experiment are detailed in the corresponding figure legends. The analysts were not blinded during data assessment. All in vivo and in vitro experiments were independently repeated at least three times to ensure reproducibility. Antibodies used in this study and primers for genotyping of *EpoR*^*Cre*^ mice and *Pim1*^*fl/fl*^ mice, and quantitative real-time polymerase chain reaction (qRT-PCR) were listed in Supplementary Data 3.

### Genotyping and blood parameter analysis

For genotyping, genomic DNA was extracted using KAPA Express Extract kit (KAPA Bio system, KK7103). AccuPrime Taq DNA Polymerse System (invitrogen, 12339-016) was used for PCR amplification. Primers for genotyping were listed in Supplementary Data 3. For blood parameter analysis, peripheral blood of indicated mice was analyzed using an advia120 hematology analyzer.

### Cell culture

The detailed in vitro human erythroid culture system has been described previously^56–58^. CD34^+^ cells were isolated from human umbilical cord blood by positive selection using a magnetic-activated cell sorting (MACS) system, following the manufacturer’s protocol. The cells were cultured in a three-phase system using a base medium consisting of Iscove’s Modified Dulbecco’s Medium (IMDM) supplemented with 2% human peripheral blood plasma, 3% human AB serum, 200 μg/mL holo-human transferrin, 3 IU/mL heparin, and 10 μg/mL insulin. Phase 1 (Days 0–6): CD34^+^ cells were seeded at a density of 1 × 10^5^ cells/mL and maintained in the base medium supplemented with 10 ng/mL stem cell factor (SCF), 1 ng/mL interleukin-3 (IL-3), and 3 IU/mL erythropoietin (EPO). Phase 2 (Days 7–11): IL-3 was withdrawn from the culture medium, while SCF and EPO were retained. Phase 3 (Days 12–21): On day 11, the cell concentration was adjusted to 1 × 10^6^ cells/mL and further increased to 5 × 10^6^ cells/mL on day 15. During this phase, cells were cultured in the base medium containing 3 IU/mL EPO, with transferrin concentration increased to 1 mg/mL. All cultures were maintained at 37 °C in a humidified 5% CO_2_ atmosphere. K562 and HEL cell lines (obtained from American Type Culture Collection) were cultured in RPMI1640 medium containing 15% FBS.

### shRNA mediated gene knockdown

As described previously^13,56,57,59,60^, shRNAs against the targeted gene of PIM1 were cloned into pLKO.1 vector. The target sequences were CGAAGAAATCCAGAACCATCC and ACATCCTTATCGACCTCAATC. Lentiviral particles were generated by co-transfecting HEK 293 T cells (obtained from American Type Culture Collection) with the packaging plasmid pCMV8.9, the envelope plasmid pUC.MDG, and the pLKO.1 vector encoding shRNA targeting either luciferase (control) or PIM1. At 48 h post-transfection, the supernatant containing viral particles was harvested, filtered through a 0.2 µm cellulose acetate membrane, and concentrated via ultracentrifugation (53,000 × *g*). The pellet from 200 mL supernatant was resuspended in 200 µL PBS and stored at –80 °C. For knockdown, 1 × 10^6^ CD34^+^ cells at day 2 were transduced with ~30 µL lentiviral supernatant (~40 × 10^6^ TU, MOI = 40). The cell-virus mixture was centrifuged at 1500 × *g* for 2 h (room temperature) to enhance viral contact, followed by incubation for 16 h at 37 °C, 5% CO_2_. Cells were then washed, resuspended in fresh medium, and cultured for 24 h before puromycin selection (1 µg/mL). Untransduced cells were eliminated within 48 h of puromycin treatment.

### Quantitative real-time PCR (qRT-PCR) analysis

As described previously^56,59–61^, total RNA was extracted from 5 × 10^5^ cells using the RNeasy Mini Kit (QIAGEN, 74104). RNA concentrations were assessed using an NanoDrop spectrophotometer (ThermoFisher Scientific). First-strand cDNA synthesis was performed using Oligo(dT) primers and the SuperScript III Reverse Transcriptase kit (Invitrogen, 18080044) following the manufacturer’s protocol. Quantitative PCR was carried out using POWER SYBR Green Master Mix (Applied Biosystems, 4367659) with the primer pairs listed in Supplementary Data 3. Primer specificity was verified by single-peak melt curve analysis. All reactions were performed in triplicate, with GAPDH serving as the endogenous control for normalization. Relative gene expression was calculated using the 2^−ΔΔCt^ method.

### Western blotting analysis

Cells were harvested, washed with ice cold 1× PBS, and lysed in RIPA buffer (APPLYGEN, C1503 + ) supplemented with protease inhibitor cocktail (Roche, 11836170001) for 30 min on ice. Cell lysates were clarified by centrifugation at 12,000 × *g* for 15 min at 4 °C. The concentrations of the protein samples were measured using the BCA protein assay kit (APPLYGEN, P1511). As described previously^3,62^, 4–30 μg of total protein was resolved by 10% SDS-polyacrylamide gel electrophoresis and subsequently transferred to nitrocellulose membranes (Bio-Rad). Membranes were blocked with 5% non-fat dry milk in TBST (Tris-buffered saline with 0.1% Tween-20) for 1 h at room temperature, followed by incubation with primary antibodies overnight at 4 °C. After washing, membranes were probed with horseradish peroxidase (HRP)-conjugated secondary antibodies (Proteintech, RGAU011) for 1 h at room temperature. Protein bands were visualized using enhanced chemiluminescence (ECL) substrate (Pierce, 32106) and imaged with a ChemiDoc imaging system (Bio-Rad) or Azure cSeries C600 system (Azure Biosystems).

### Colony forming assay

For human erythroid cells, sorted erythroid progenitor cells were diluted at a density of 200 cells in 1 mL of MethoCult H4330 medium (STEMCELL Technologies) for CFU-E colony forming analysis or H4434 medium (STEMCELL Technologies) for BFU-E colony forming analysis, and incubated at 37 °C in a humidified atmosphere with 5% CO_2_. The colony numbers of CFU-E and BFU-E were counted 7 days and 14 days later, respectively^31^. For mouse erythroid cells, mouse bone marrow (BM) cells were also plated in triplicate at a density of 50,000 cells per well in 1 mL of MethoCult M3334 (Stemcell Technologies, #03334) or M3434 (Stemcell Technologies, #03434)^63^. These plates were incubated under identical conditions, with CFU-E and BFU-E colonies counted on Days 3 and 7, respectively^63^.

### Flow cytometry analysis

Human erythroid progenitors: 10^5^ cells (day 6) were washed and suspended in 25 μL PBS/2% FBS/2 mM EDTA and stained with IL-3R-PE-Cy7, GPA-APC, CD34-PE, and CD36-FITC for 30 min at 4 °C^31^. Human terminal erythropoiesis: terminal differentiated erythroblasts (day 7 to day 17) were analyzed using GPA-PE, Band3-FITC, and α4-integrin-APC with identical conditions^3^. Mouse erythroid progenitors: lineage depleted BM cells were prepared by incubating with biotinylated lineage antibodies (Gr1/CD11b/Ter119/CD3e/B220) followed by anti-biotin microbeads (Miltenyi Biotec). Enriched Lineage^-^ cells (2 × 10^5^) were suspended in 50 μL PBS/2% FBS/2 mM EDTA and stained with CD16/32-BV421, CD71-FITC, CD34-APC, Sca1-APC, c-Kit-APC-Cy7, CD41-eFluor450 and Streptavidin-PerCP for 30 minutes at room temperature^35^. Mouse terminal erythropoiesis in vivo: whole BM cells (10^6^) were suspended in 50 μL PBS/2% FBS/2 mM EDTA and labeled with Ter119-V450, CD44-APC, CD45/CD11b/Gr1-APC-Cy7 for 30 min at room temperature^36^. Mouse terminal erythropoiesis in vitro: cells (10^5^) were suspended in 50 μL PBS/2% FBS/2 mM EDTA and labeled with Ter119-V450 and CD71-FITC for 30 min at room temperature. Hoechst33342 was used for erythroid enucleation analysis (10^5^ cells in 25 μL PBS/2% FBS/2 mM EDTA at 37 °C for 20 min). Annexin V was used for cell apoptosis analysis (10^5^ human cells in 25 μL PBS/2% FBS/2 mM EDTA or 10^6^ mouse cells in 50 μL PBS/2% FBS/2 mM EDTA at room temperature for 15 min). Viability was assessed using 7-AAD (5 min on ice). For intracellular staining of Ki67, mouse BM cells were initially labeled with specific markers of terminal erythroid differentiation (CD45, Gr1, CD11b, Ter119, CD44), followed by fixation in 4% paraformaldehyde (15 minutes) and permeabilization with 0.1% Triton X-100 (15 min). and stained with anti-Ki67-FITC for 1 h at room temperature. All samples were acquired within 1 h on an LSR Fortessa flow cytometer (Becton Dickinson) using FACSDiva 6.1.2.

### Amnis imaging flow cytometry analysis

The erythroid cells were stained with PE-conjugated anti-mouse CD71 and Hoechst33342 at 37 °C for 20 min. After washing for 2 times with PBS, the cells were analyzed with Amnis ImageStream Mark II instrument at 60× magnification. IDEAS software was used to analyze Imaging Flow data.

### Cytospin preparation and May-Grunwald Giemsa Staining

Cytospin was prepared on coated slides with 5 × 10^4^ cells by Thermo Scientific Shandon 4 Cytospin at 400 × *g*, 3 min. The air-dried slides were initially stained with May-Grunwald solution (Sigma MG500) for a duration of 5 min. Subsequently, they were rinsed in 40 mM Tris buffer (pH 7.4) for 90 s to remove excess stain. The slides were then subjected to staining with Giemsa solution (diluted 10-fold with water) (Sigma GS500) for 15 min. Finally, the slides were thoroughly rinsed twice with water to ensure complete removal of residual stains. The stained slides were examined under a Leica DM2000 inverted microscope, and images were captured for subsequent analysis.

### Transmission electron microscopy (TEM) analysis

TEM images were obtained by a general protocol. Erythroid cells on day 15 were washed twice by PBS and then fixed with 2.5% glutaraldehyde. Next, the cells were fixed with 1% osmium tetroxide followed by dehydration, embedding, sectioning, and staining for TEM images.

### Confocal microscopy analysis

For mitochondria staining, the cultured erythroid cells at indicated days were rinsed by PBS and treated with staining solution (100 nM) of Mito-tracker Red (Beyotime Biotechnology) for about 15 min; For LC3B tracking, the coding sequencing of GFP-LC3B were cloned into pLVX-EF1a-Puro vector and the vector were co-transfected with pLKO.1-shRNA vectors into CD34^+^ cells on day 2; For FM4-64 staining, cells were washed with PBS twice and stained with FM4-64 working solution (10 μM) for 1 min on ice. For F-actin staining, cells were washed with PBS and then fixed with 3.7% formaldehyde fixative for 10 min at room temperature, permed with 0.1% Triton X-100 solution for 10 min at room temperature. Subsequently, the cells were stained with Actin-tracker Green working solution (1: 100 diluted) for 1 h at room temperature. Hoechst33342 was used for staining nuclear when needed. All the fluorescent images were captured with a confocal microscopy (Zeiss 780).

### Phosphorylation proteomics analysis

Sorted Ortho from day 15 of culture were lysed in a buffer containing 8 M urea, 1% protease inhibitor, and 1% phosphatase inhibitor. Cell debris was removed by centrifugation at 4 °C, 12,000 × *g* for 10 min. The supernatant was transferred to a new tube, and protein concentration was determined using a BCA kit (Beyotime Biotechnology). Proteins were reduced with 5 mM dithiothreitol at 56 °C for 30 min, followed by alkylation with 11 mM iodoacetamide at room temperature for 15 min in the dark. Urea concentration was then diluted to below 2 M. Sequencing-grade trypsin (Promega) was added at a 1:50 (trypsin: protein) mass ratio for overnight digestion at 37 °C, followed by a second addition at a 1:100 ratio for 4 h. Peptides were desalted using Strata X C18 (Phenomenex) resin, lyophilized, and labeled according to the TMT kit (Thermo Fisher Scientific) protocol. Labeled peptides were fractionated by high-pH reverse-phase High Performance Liquid Chromatography (HPLC) using a Thermo Betasil C18 column. After elution and drying, peptides were resuspended in 0.1% formic acid and separated on an EASY-nLC 1000 system coupled to a Q Exactive^TM^ mass spectrometer. Data-dependent acquisition mode was used for MS/MS analysis, with specific parameters set for precursor and fragment ion tolerance, fixed modifications, variable modifications (methionine oxidation, N-terminal acetylation, serine/threonine/tyrosine phosphorylation), and TMT-6plex quantification. Database searching was performed with Maxquant software against the SwissProt Human database supplemented with common contaminants and a decoy database for FDR calculation. Mass error and peptide length distribution were used to assess data quality. Differential phosphorylation was assessed using Student’s *t* test with a threshold of *P* < 0.05 and fold change >1.5. Pathway analysis was conducted using the Gene Ontology analysis. Heatmaps and volcano plots were generated using R software.

### Statistics and reproducibility

Flow cytometry data were analyzed using FlowJo software, while ImageJ was employed for analyzing band signal intensities. Intergroup comparisons were performed using unpaired student’s *t* tests for two groups, One-way ANOVA for multiple groups or Two-way ANOVA for growth curves, as specified in the respective figure legends. All statistical analyses were conducted using GraphPad Prism 10 software with statistical significance set at *P < 0.05, **P < 0.01, ***P < 0.001. Data are presented as mean ± SEM from at least three independent biological replicates per experimental group. Sample sizes were determined to ensure adequate statistical power while considering experimental feasibility and resource availability. All experiments were independently replicated a minimum of two times.

### Reporting summary

Further information on research design is available in the Nature Portfolio Reporting Summary linked to this article.

## Supplementary information

Supplementary Information

Description of Additional Supplementary Files

Supplementary Data 1

Supplementary Data 2

Supplementary Data 3

Supplementary Data 4

Reporting Summary

## Data Availability

All statistical datasets supporting the findings of this study are provided as Supplementary Data 4 accompanying this manuscript. Supplementary data and raw data for western blot (Supplementary Fig. 9) can be found in the Supplementary Information. Antibody specifications and primer sequences have been cataloged in Supplementary Data 3. Quantitative phosphoproteomics data are presented in Supplementary Data 1, with the raw mass spectrometry files deposited in the ProteomeXchange Consortium (Dataset PXD067668) through the iProX partner repository. Additional datasets generated during this study are available from the corresponding author upon reasonable request.
