# Supplementary material for: The oncogene protein kinase PIM1 regulates mammalian erythroblast enucleation
Source: Commun Biol. 2025 Oct 15;8:1473. doi: 10.1038/s42003-025-08869-0 (PMC12528402; doi:10.1038/s42003-025-08869-0)
Supplement: Supplementary file 1 — Supplementary Information [file 42003_2025_8869_MOESM1_ESM.pdf]

SUPPLEMENTARY FIGURES AND TABLES

Supplementary Figure 1

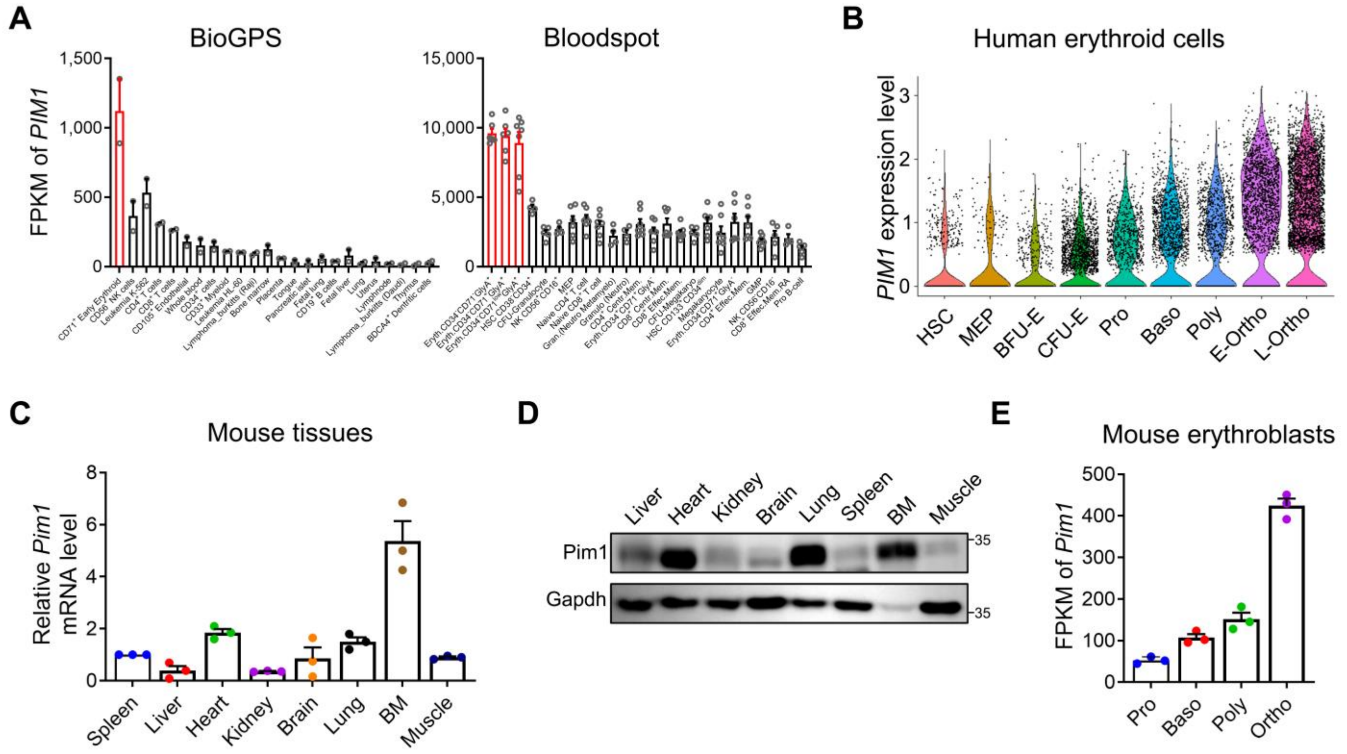

Supplementary Figure 1. PIM1 expression in human and mouse tissues and cells. (A) *PIM1* expression in different human tissue and cell types according to human tissue expression database of gene-annotation portal BioGPS and human healthy and malignant hematopoietic tissue expression database Bloodspot. **(B)** Expression level of *PIM1* in HSPCs, erythroid progenitors and erythroblasts according to the single cells RNA-Seq data of human BM. **(C)** qRT-PCR analysis showing the expression of *Pim1* in indicated tissues of mice. **(D)** Western blotting analysis showing the expression of Pim1 in in indicated tissues of mice. **(E)** RNA-seq data revealing the gene expression levels of *Pim1* at distinct stages of mouse Pro, Baso, Poly and Ortho. Data were presented as mean ± SEM. FPKM: Fragments Per Kilobase Million. HSC: Hematopoietic Stem Cell. MEP: Megakaryocyte-Erythroid Progenitor.

Supplementary Figure 2

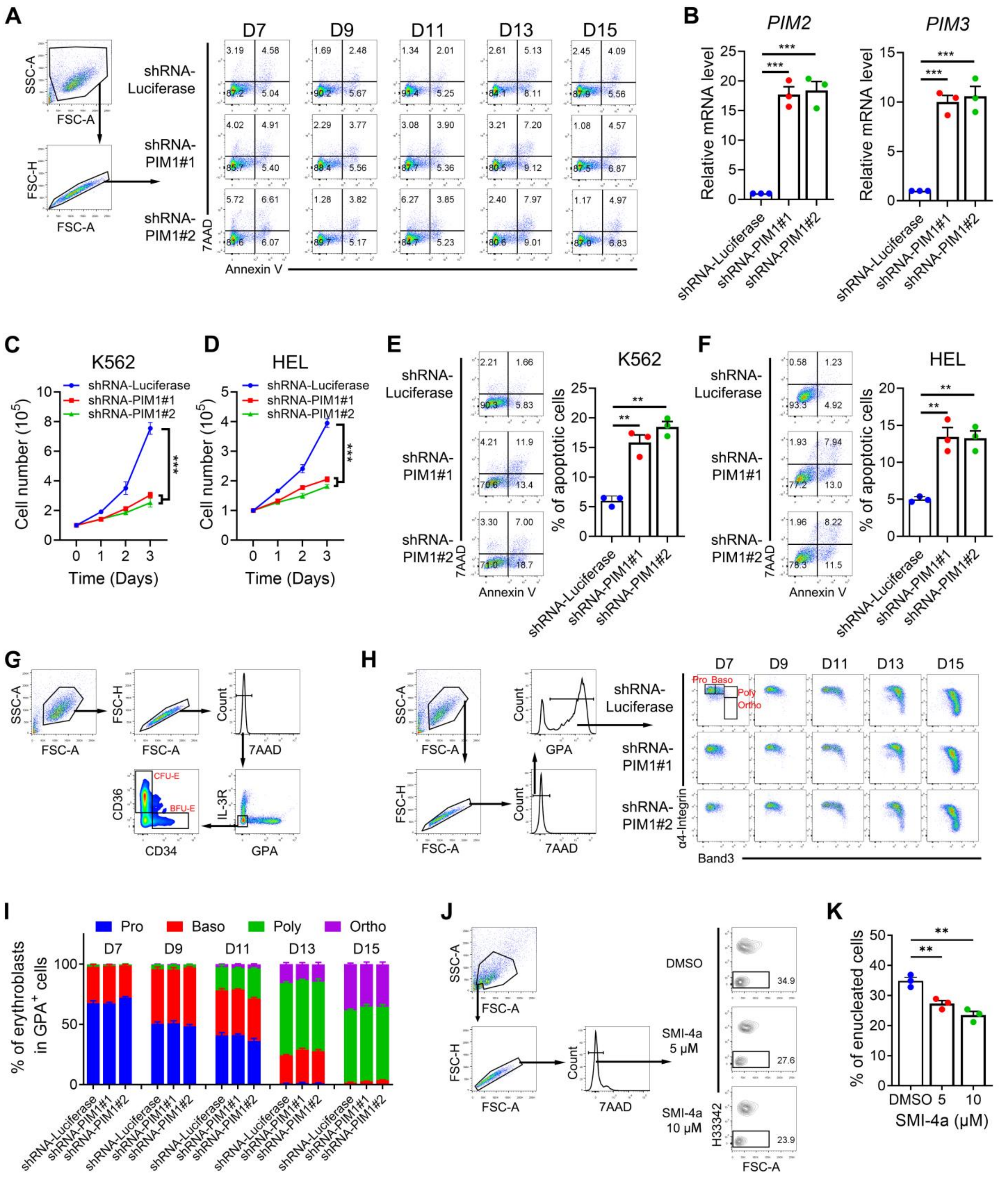

Supplementary Figure 2. The effect of PIM1 on cell apoptosis, terminal erythropoiesis and enucleation. (A) Flow cytometry analyses showing apoptotic rate of cultured human erythroid cells transfected with shRNA-Luciferase or shRNA-PIM1 at indicated day. **(B)** Bar plots showing the mRNA level of *PIM2* and *PIM3* in PIM1 knockdown and control cells. **(C, D)** Growth curve of K562 cells and HEL cells transfected with shRNA-Luciferase or shRNA-PIM1. **(E, F)** Flow cytometry analyses and quantitative results showing apoptotic rate of K562 cells and HEL cells transfected with shRNA-Luciferase or shRNA-PIM1. **(G)** Gating strategy for human erythroid progenitors. **(H, I)** Flow cytometry analyses and quantification results showing the percentage of different stages of erythroblasts defined by the expression

of α4-integrin and Band3 at the indicated days. **(J, K)** Flow cytometry analyses and quantitative results showing the enucleation rate of day 13 erythroid cells after treated with DMSO or SMI-4a for 2 days. Data were presented as mean ± SEM. *P* values were determined by either One-way ANOVA (**B, E, F, I, K**) or Two-way ANOVA (**C, D**). 7AAD: 7-Aminoactinomycin D; H33342: Hoechst33342. $^{**}P < 0.01$, $^{***}P < 0.001$.

Supplementary Figure 3

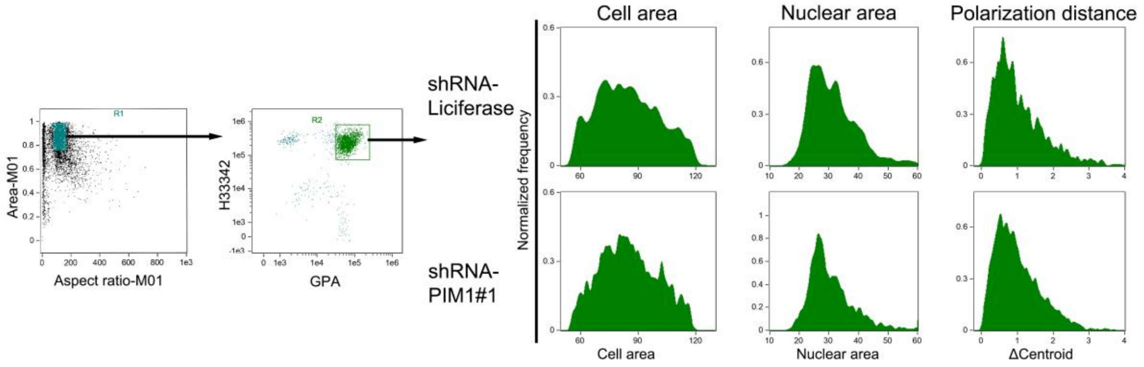

Supplementary Figure 3. Gating procedure for the cell area, nuclear area and polarization distance analysis using ImageStream data. Day 17 cultured human erythroid cells were stained with GPA and Hoechst33342. $GPA^{+}Hoechst33342^{+}$ cells were used for further analysis. Cell area and nuclear area were analyzed with the features of area of bright field and Hoechst33342, respectively. Polarization distance were calculated by the feature Δ centroid which meant the distance from the central of the cell to the central of its nucleus.

Supplementary Figure 4

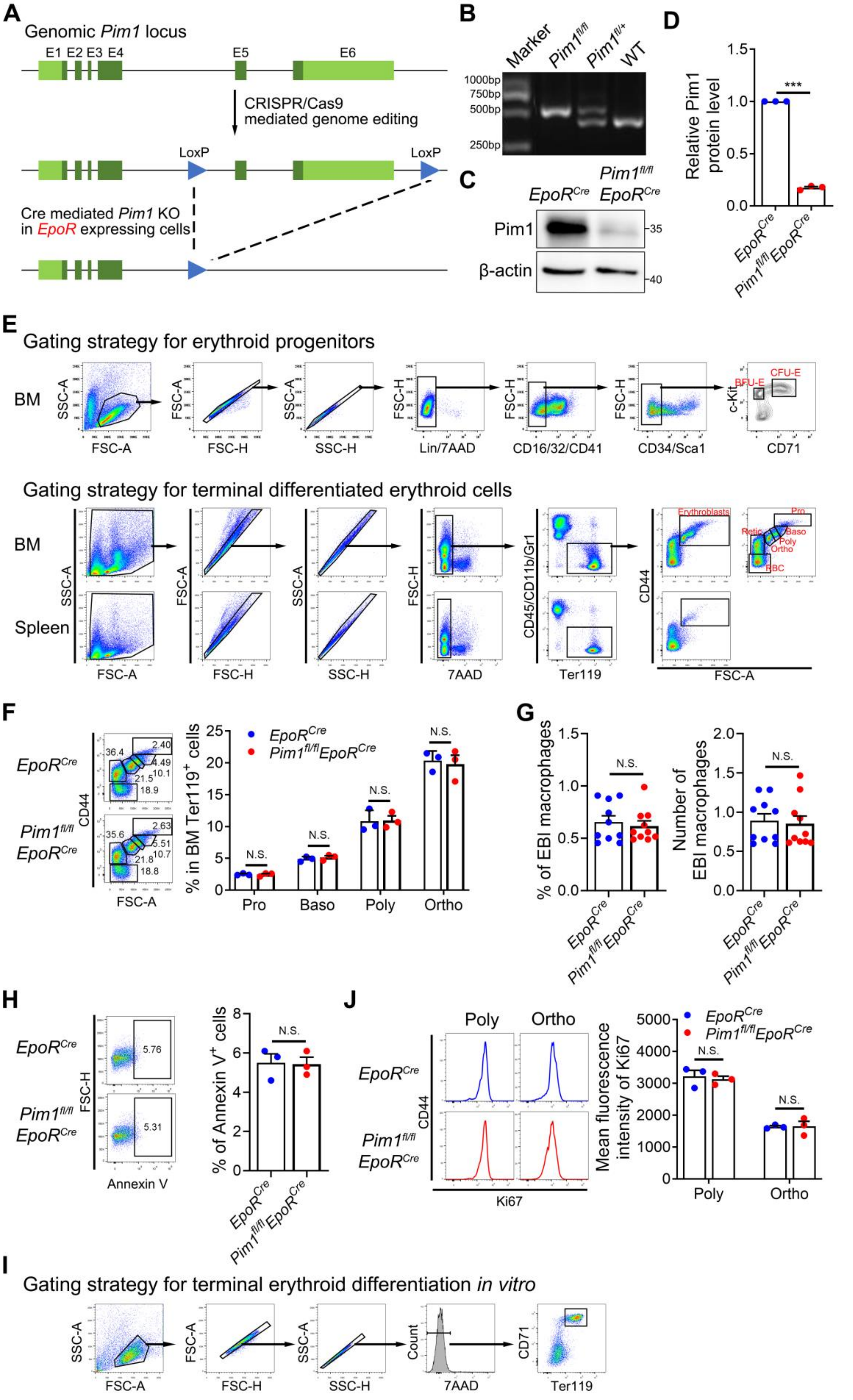

Supplementary Figure 4. PIM1 deletion did not affect the terminal erythroid differentiation and apoptosis of erythroid cells in mice. (A) Schematic model for generation of *Pim1*-loxP mice and *Pim1*$^{fl/fl}$*EpoR*^{Cre} mice. **(B)** PCR analysis showing the genotypes of *Pim1*-loxP mice. **(C, D)** Western blotting analyses and statistical results showing the

expression of Pim1 in BM Ter119$^+$ cells of the *Pim1$^{fl/fl}$EpoR^{Cre}* mice. **(E)** Gating strategy for mouse erythroid progenitors and terminal differentiated erythroid cells *in vivo*. **(F)** Representative plots of CD44 versus FSC-A of BM Ter119$^+$ cells revealing different staged erythroid cells and their quantification. **(G)** Bar plots showing the percentage and number of EBI macrophages in BM of *Pim1$^{fl/fl}$EpoR^{Cre}* mice and controls. N=10. **(H)** Flow cytometry analyses and quantitative results showing apoptotic rate of erythroblasts in *Pim1$^{fl/fl}$EpoR^{Cre}* mice and control group mice. **(I)** Gating strategy for mouse terminal erythroid differentiation *in vitro*. **(J)** Flow cytometry analyses and statistical results displaying the mean fluorescence intensity of Ki67 in Poly and Ortho of *Pim1$^{fl/fl}$EpoR^{Cre}* mice and controls. Data were presented as mean ± SEM. *P* values were determined by student's *t* test. E (in panel A): exon; WT: wild type; BM: bone marrow; FSC: forward scatter; SSC: Side Scatter; EBI: erythroblastic island; *P* values were determined by student's *t* test. N.S.: no statistic different. ***$P < 0.001$.

Supplementary Figure 5

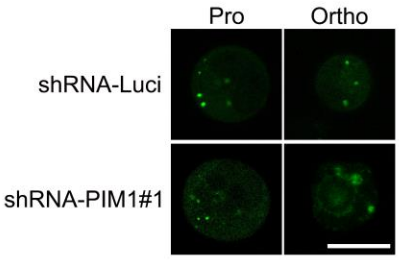

Supplementary Figure 5. PIM1 knockdown led to the increase of autophagy in Ortho. Fluorescent images showing the autophagosomes in indicated human cultured erythroid cells transfected with shRNA-Luciferase or shRNA-PIM1. Autophagosomes were visualized by the aggregation of GFP-LC3B expressed in cells. Scale bar = 10 μm.

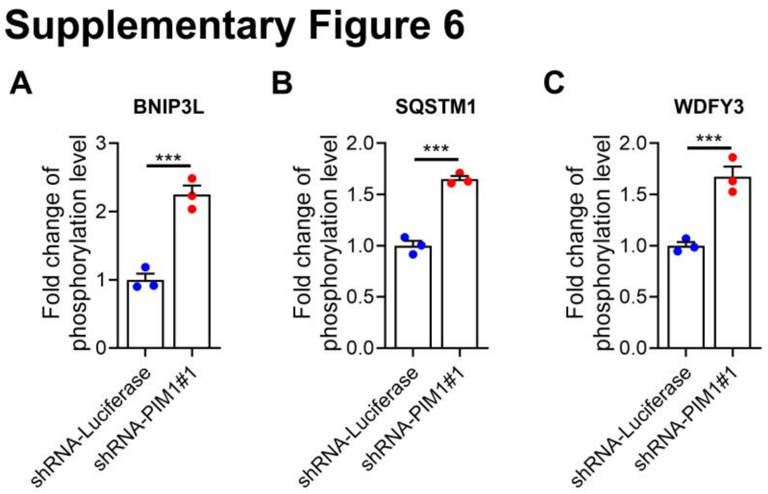

Supplementary Figure 6. Fold change of up phosphorylated proteins that related to mitochondrial quality control and autophagy in PIM1 knockdown Ortho. (A) BNIP3L. **(B)** SQSTM1. **(C)** WDFY3. Data were presented as mean ± SEM. *P* values were determined by student's *t* test. ***$P < 0.001$.

Supplementary Figure 7

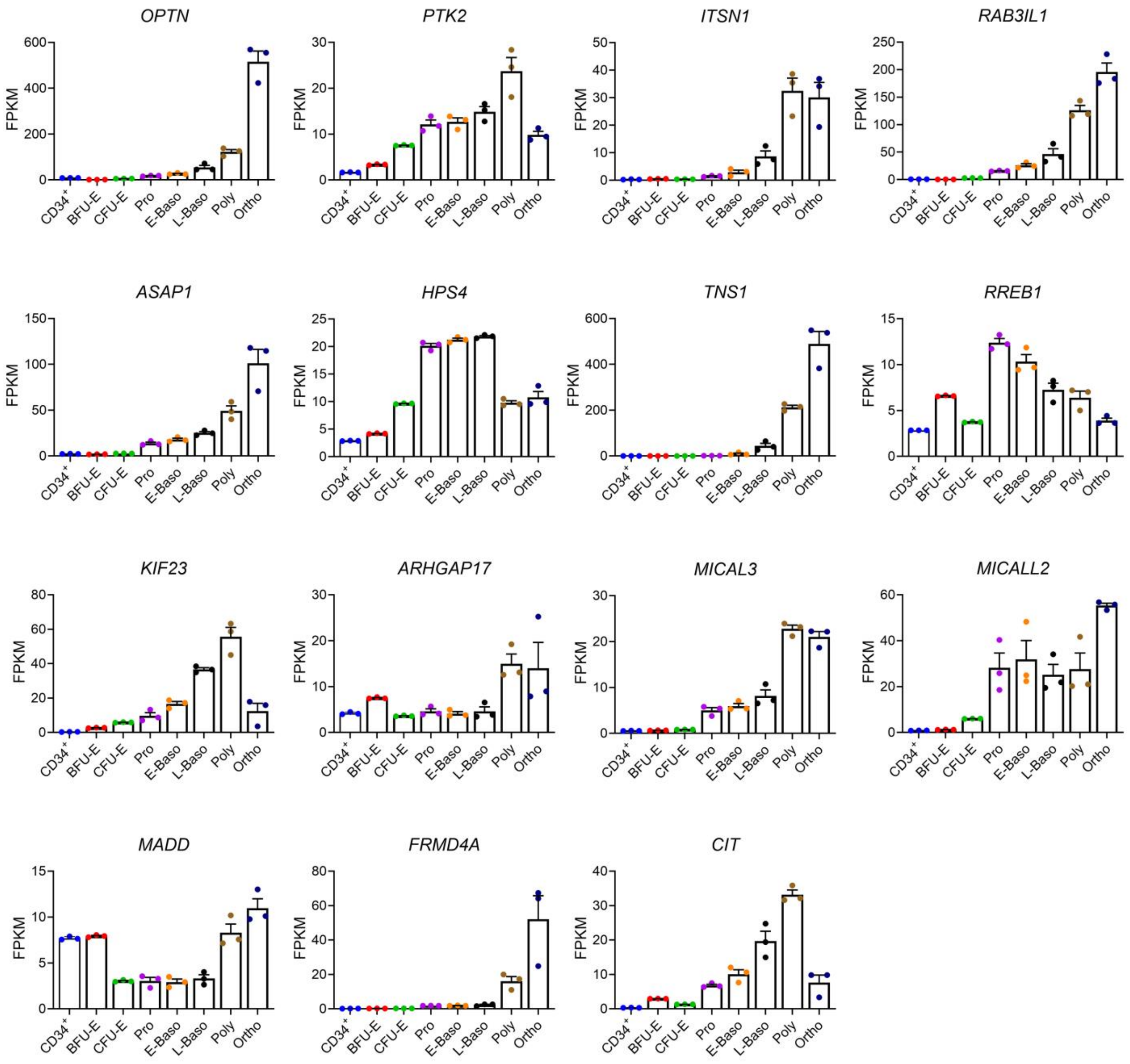

Supplementary Figure 7. Expression of GTPase related proteins that down phosphorylated in PIM1 knockdown Ortho. Data were presented as mean ± SEM.

Supplementary Figure 8

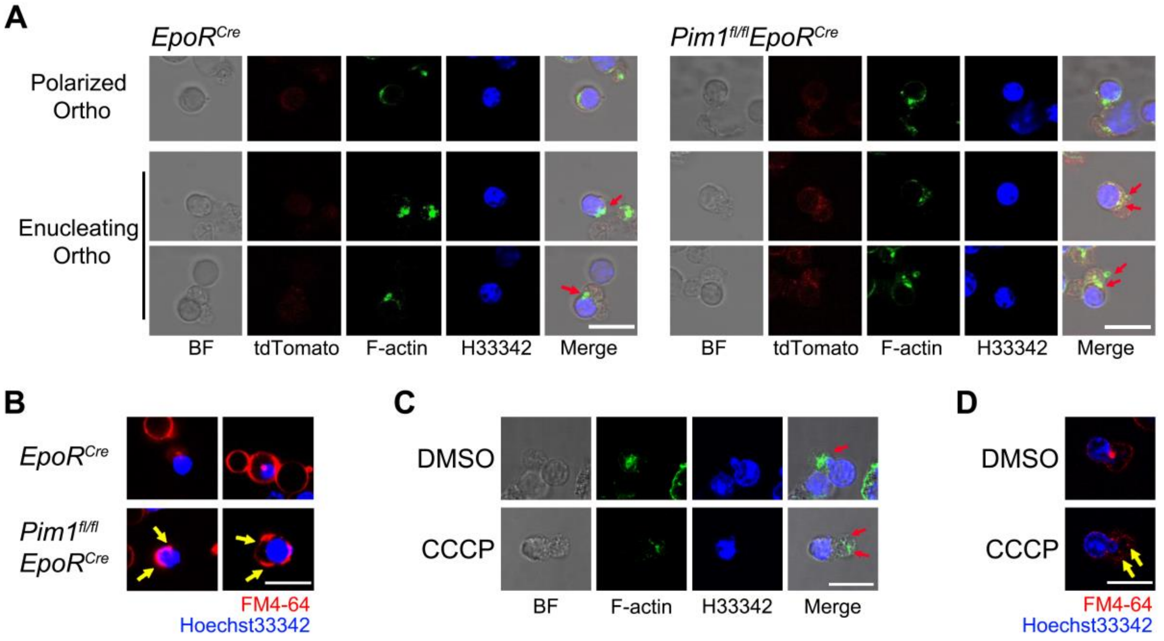

Supplementary Figure 8. PIM1 controlled F-actin network and vesicle trafficking in Ortho of mice. (A) Fluorescent images showing the localization of F-actin in polarized and enucleating Ortho of *Pim1$^{fl/fl}$EpoR^{Cre}* mice and controls. Scale bar = 10 µm. **(B)** Fluorescent images demonstrating the internalization of membrane-impermeable dye FM4-64 in enucleating Ortho of *Pim1$^{fl/fl}$EpoR^{Cre}* mice and controls. Scale bar = 10 µm. **(C)** Fluorescent images showing the localization of F-actin in enucleating Ortho of after treated with 10 µM CCCP or DMSO for 2 days. Scale bar = 10 µm. **(D)** Fluorescent images demonstrating the internalization of FM4-64 in enucleating Ortho after treated with 10 µM CCCP or DMSO for 2 days. Scale bar = 10 µm. H33342: Hoechst33342. DMSO: dimethyl sulfoxide. CCCP: Carbonyl Cyanide m-Chlorophenyl Hydrazine.

Supplementary Figure 9. Full unedited images for WB results

Fig 1C

PIM1

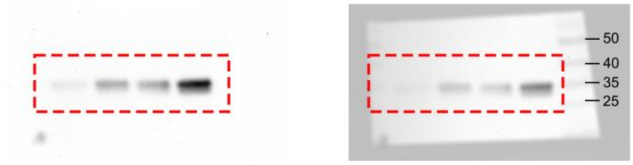

GAPDH

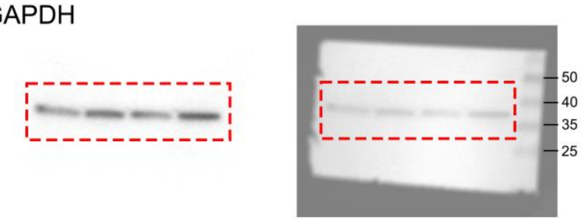

Fig 1E

PIM1

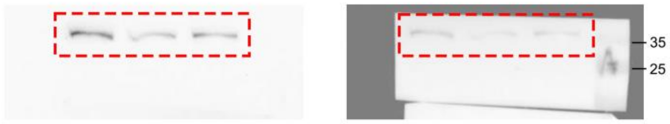

GAPDH

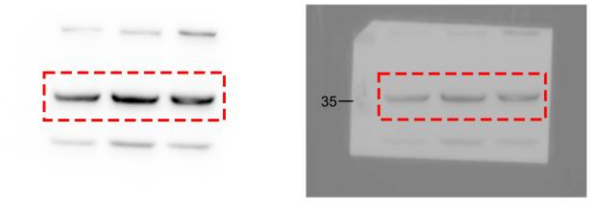

Supplementary Figure 1D

PIM1

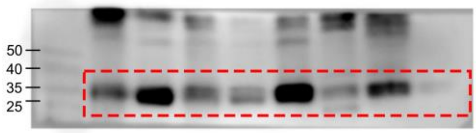

GAPDH

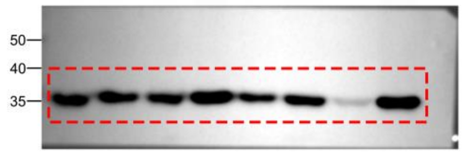

Supplementary Figure 4C

PIM1

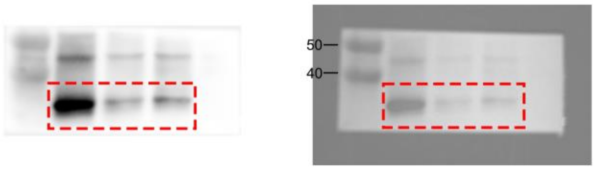

GAPDH

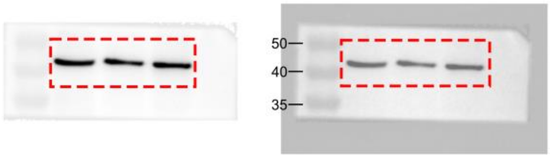

Supplementary Table 1. Top 20 expressed kinase genes in Ortho

Rank	Official Symbol	FPKM	Kinase type	Function annotation
1	*PIM1*	4426.02	Serine/Threonine Kinase	Cell survival; cell proliferation; cell cycle; anti-apoptotic.
2	*RIOK3*	2814.5	Serine/Threonine Kinase	Innate immune response.
3	*MAP2K3*	2468.4	Serine/Threonine Kinase	Proliferation, differentiation, transcription regulation and development.
4	*EIF2AK1*	2242.09	Ser/Thr protein kinase family	The phosphorylation of EIF2S1 at Ser-48 and Ser-51.
5	*STRADB*	1491.98	serine/threonine protein kinase	Regulates cell polarity and energy-generating metabolism.
6	*MKNK2*	1139.75	Serine/Threonine Kinase	Antileukemic responses; arsenic trioxide As_2O_3-dependent apoptosis.
7	*TRIB3*	1013	CAMK Ser/Thr protein kinase family	Transcriptional activation activity.
8	*WNK1*	901.547	Ser/Thr protein kinase family	Actin cytoskeletal reorganization; sodium-chloride cotransporter.
9	*STK11*	838.219	CAMK Ser/Thr protein kinase family	Metabolism; cell polarity; apoptosis and DNA damage response.
10	*PIM2*	790.515	Serine/Threonine Kinase	Pro-apoptotic protein; survival signaling; cell cycle.
11	*STK40*	611.555	CAMK Ser/Thr protein kinase family	May be a negative regulator of NF-kappa-B and p53-mediated gene transcription.
12	*STK17A*	557.43	CAMK Ser/Thr protein kinase family	Apoptosis; cellular reactive oxygen species.
13	*PTK2B*		Tyr protein kinase family. FAK subfamily.	Calcium-induced regulation of ion channels; activation of the map kinase signaling pathway.
14	*MARK3*	439.072	CAMK Ser/Thr protein kinase family	Phosphorylation of microtubule-associated tau proteins.
15	*BMP2K*	437.837	Ser/Thr protein kinase family	May be involved in osteoblast differentiation.
16	*MAST3*	433.222	AGC Ser/Thr protein kinase family	Related with Inflammatory Bowel Disease 6.
17	*DUSP1*	380.652	protein-tyrosine phosphatase family	By oxidative stress and heat shock.
18	*DYRK3*	372.68	CMGC Ser/Thr protein kinase family	A central dissolvase of membraneless organelles.
19	*STK17B*	331.726	CAMK Ser/Thr protein kinase family	Phosphorylates myosin light chains. Acts as a positive regulator of apoptosis.
20	*ULK1*	324.731	Ser/Thr protein kinase family	Autophagy; autophagophores.

Supplementary Table 2. Function annotation of the top 25 expressed genes in Ortho

Rank	Official Symbol	Function annotation
1	*HBB*	Subunit of adult HbA; Blood coagulation; cellular oxidant detoxification; oxygen transport.
2	*HBG2*	Subunit of fetal HbF.
3	*HBA2*	Subunit of Hb; Bicarbonate transport; cellular oxidant detoxification; hydrogen peroxide catabolic process.
4	*HBA1*	Subunit of Hb; Positive regulation of cell death; oxygen transport; cellular oxidant detoxification.
5	*HBG1*	Subunit of fetal HbF.
6	*FTL*	Storing iron in a soluble, non-toxic, readily available form. Important for iron homeostasis.
7	*ALAS2*	An erythroid-specific mitochondrially located enzyme, catalyzing the first step of porphyrin biosynthesis.
8	*UBB*	Cell cycle; lysosomal degradation; kinase modification; endocytosis.
9	*SLC4A1*	Functions both as a transporter that mediates electroneutral anion exchange across the cell membrane and as a structural protein.
10	*FTH1*	Stores iron in a soluble, non-toxic, readily available form. Important for iron homeostasis.
11	*BSG*	Plays an important role in targeting the monocarboxylate transporters SLC16A1, SLC16A3 and SLC16A8 to the plasma membrane. Outgrowth of astrocytic processes.
12	*SLC25A37*	Mitochondrial iron transporter; heme biosynthesis.
13	*TMCC2*	Involved in the regulation of the proteolytic processing of the amyloid precursor protein (APP) possibly also implicating APOE.
14	*SLC25A39*	Normal heme biosynthesis
15	*SLC2A1*	Facilitative glucose transporter.
16	*RBM38*	Cell cycle arrest.
17	*AHSP*	A chaperone to stabilize alpha-hemoglobin during normal erythroid cell development.
18	*NCOA4*	Enhances the androgen receptor transcriptional activity in prostate cancer cells.
19	*BLVRB*	Broad specificity oxidoreductase that catalyzes the NADPH-dependent reduction of a variety of flavins.
20	*OAZ1*	Induced by a ribosomal frameshifting mechanism in response to increased levels of intracellular polyamines.
21	*BCL2L1*	Anti-apoptosis.
22	*FKBP8*	Anti-apoptosis.
23	*PIM1*	Cell cycle; cell survival; cell proliferation; anti-apoptosis.
24	*DCAF12*	Protein degradation.
25	*BPGM*	Regulating hemoglobin oxygen affinity by controlling the levels of its allosteric effector 2,3-bisphosphoglycerat.

Supplementary Table 3. Functional annotation of the down phosphorylated GTPase related proteins in PIM1 KD Ortho

Rank	Official Symbol	Function annotation
1	ITSN1	Regulates the formation of clathrin-coated vesicles, stimulates actin nucleation
2	ASAP1	Regulates membrane trafficking and cytoskeleton remodeling.
3	ARHGAP17	Acts as a GTPase activator, regulates the activity of CDC42.
4	MICAL3	Regulates actin filament disassembly and vesicle trafficking.
5	MADD	Regulates cell apoptosis; Converts GDP-bound inactive form of RAB3A, RAB3C and RAB3D to the GTP-bound active forms.
6	OPTN	An autophagy receptor for damaged mitochondria, regulates the maintenance of the Golgi complex, membrane trafficking and actin network.
7	CIT	Putative RHO/RAC effector, initiates efficient cytokinesis with KIF14
8	FRMD4A	Regulates cell polarization, adherens junctions, and the formation of linear actin.
9	MICALL2	Effector of small Rab GTPases, Regulates the reorganization of actin and the endocytosis recycling of proteins.
10	PTK2	Regulates autophagy, actin dynamics and vesicle trafficking.
11	RAB3IL1	GEF for RAB3A, activates RAB8A and RAB8B to regulate vesicle trafficking.
12	HPS4	GEF for RAB32 and RAB38, important for the formation of endosomal-lysosomal organelles.
13	KIF23	Serves as a microtubule-dependent and Rho-mediated signaling required for the myosin contractile ring formation.
14	RIPOR1	Effector of small Rho GTPases， regulates the maintenance of the Golgi complex.
15	TNS1	Regulates cell polarization and migration, crosslinks actin filaments.

Supplementary Table 4. GO analysis of the down phosphorylated GTPase related proteins in PIM1 KD cells

Term	Genes	*P* Value
GO:0006887~exocytosis	ITSN1, MICAL3, RAB3IL1	0.0039985
GO:0034067~protein localization to Golgi apparatus	RIPOR1, OPTN	0.0152972
GO:0043001~Golgi to plasma membrane protein transport	ASAP1, OPTN	0.025123
GO:1903955~positive regulation of protein targeting to mitochondrion	MICALL2, HPS4	0.0273775
GO:0010634~positive regulation of epithelial cell migration	RREB1, PTK2	0.0281279
GO:0032467~positive regulation of cytokinesis	KIF23, CIT	0.0311241
GO:0032956~regulation of actin cytoskeleton organization	ARHGAP17, CIT	0.0771918
