# Supplementary material for: The oncogene protein kinase PIM1 regulates mammalian erythroblast enucleation
Source: Commun Biol. 2025 Oct 15;8:1473. doi: 10.1038/s42003-025-08869-0 (PMC12528402; doi:10.1038/s42003-025-08869-0)
Supplement: Supplementary file 2 — Description of Additional Supplementary Files [file 42003_2025_8869_MOESM2_ESM.docx]

Description of Additional Supplementary Files

**File name:** Supplementary Data 1

**Description:** The phosphorylation proteomics data produced in this study.

**File name:** Supplementary Data 2

**Description:** The differentially phosphorylated phosphosites.

**File name:** Supplementary Data 3

**Description:** Antibodies and Primers.

**File name:** Supplementary Data 4

**Description:** Source data related to this paper.
