# Supplementary material for: The oncogene protein kinase PIM1 regulates mammalian erythroblast enucleation
Source: Commun Biol. 2025 Oct 15;8:1473. doi: 10.1038/s42003-025-08869-0 (PMC12528402; doi:10.1038/s42003-025-08869-0)
Supplement: Supplementary file 7 — Reporting Summary [file 42003_2025_8869_MOESM7_ESM.pdf]

nature portfolio

Corresponding author(s): Shijie Zhang and Xiuli An

Last updated by author(s): Aug 16, 2025

Reporting Summary

Nature Portfolio wishes to improve the reproducibility of the work that we publish. This form provides structure for consistency and transparency in reporting. For further information on Nature Portfolio policies, see our Editorial Policies and the Editorial Policy Checklist.

Statistics

For all statistical analyses, confirm that the following items are present in the figure legend, table legend, main text, or Methods section.

n/a	Confirmed	
☐	☒	The exact sample size (*n*) for each experimental group/condition, given as a discrete number and unit of measurement
☐	☒	A statement on whether measurements were taken from distinct samples or whether the same sample was measured repeatedly
☐	☒	The statistical test(s) used AND whether they are one- or two-sided<br>*Only common tests should be described solely by name; describe more complex techniques in the Methods section.*
☒	☐	A description of all covariates tested
☒	☐	A description of any assumptions or corrections, such as tests of normality and adjustment for multiple comparisons
☐	☒	A full description of the statistical parameters including central tendency (e.g. means) or other basic estimates (e.g. regression coefficient) AND variation (e.g. standard deviation) or associated estimates of uncertainty (e.g. confidence intervals)
☒	☐	For null hypothesis testing, the test statistic (e.g. *F*, *t*, *r*) with confidence intervals, effect sizes, degrees of freedom and *P* value noted<br>*Give P values as exact values whenever suitable.*
☒	☐	For Bayesian analysis, information on the choice of priors and Markov chain Monte Carlo settings
☒	☐	For hierarchical and complex designs, identification of the appropriate level for tests and full reporting of outcomes
☐	☒	Estimates of effect sizes (e.g. Cohen's *d*, Pearson's *r*), indicating how they were calculated

Our web collection on statistics for biologists contains articles on many of the points above.

Software and code

Policy information about availability of computer code

Data collection	Commercial softwares licensed by companies were utilized: Zeiss Zen 2012 software (for LSM 780); ISX Software (for Amnis ImageStream Mark II); BD FACSDiva 6.1.2 software (for BD LSR Fortessa flow cytometer)
Data analysis	For FCS files generated by flow cytometry, data analysis was performed using FlowJo 10.8.1 software. Fluorescence images acquired by confocal microscopy were analyzed using Zeiss Zen 2012 software, while raw data from Imagestream were processed with IDEAS 4.0 software. ImageJ software was used for grayscale analysis of images. Graph pad prism 10.0 software was used for data analysis.

For manuscripts utilizing custom algorithms or software that are central to the research but not yet described in published literature, software must be made available to editors and reviewers. We strongly encourage code deposition in a community repository (e.g. GitHub). See the Nature Portfolio guidelines for submitting code & software for further information.

Data

Policy information about availability of data

All manuscripts must include a data availability statement. This statement should provide the following information, where applicable:

- Accession codes, unique identifiers, or web links for publicly available datasets
- A description of any restrictions on data availability
- For clinical datasets or third party data, please ensure that the statement adheres to our policy

The authors confirm that the data supporting the findings of this study are available within the article and its supplementary materials. For raw data, please contact corresponding author, Shijie Zhang (Shijie-zhang@zzu.edu.cn).

Research involving human participants, their data, or biological material

Policy information about studies with human participants or human data. See also policy information about sex, gender (identity/presentation), and sexual orientation and race, ethnicity and racism.

Reporting on sex and gender	Not involved.
Reporting on race, ethnicity, or other socially relevant groupings	Not involved.
Population characteristics	Not involved.
Recruitment	Not involved.
Ethics oversight	Not involved.

Note that full information on the approval of the study protocol must also be provided in the manuscript.

Field-specific reporting

Please select the one below that is the best fit for your research. If you are not sure, read the appropriate sections before making your selection.

☒ Life sciences ☐ Behavioural & social sciences ☐ Ecological, evolutionary & environmental sciences

For a reference copy of the document with all sections, see nature.com/documents/nr-reporting-summary-flat.pdf

Life sciences study design

All studies must disclose on these points even when the disclosure is negative.

Sample size	A standard sample size of three biological replicates was used in this study. This approach is well-established in the field, as triplicate replicates provide sufficient statistical power to assess reproducibility while accounting for biological variability in cell-based assays. The use of three replicates aligns with common practice in in vitro studies, ensuring reliable and interpretable results without excessive resource expenditure. While formal sample size calculations were not performed, the consistency of this methodology across peer-reviewed publications supports its validity for detecting significant experimental effects.
Data exclusions	Exclusion criteria were not needed.
Replication	All data included in this study are reproducible.
Randomization	A method for randomization was not needed since experimental groups were predetermined.
Blinding	In this study automated quantitative methods were used to avoid investigator bias.

Reporting for specific materials, systems and methods

We require information from authors about some types of materials, experimental systems and methods used in many studies. Here, indicate whether each material, system or method listed is relevant to your study. If you are not sure if a list item applies to your research, read the appropriate section before selecting a response.

Materials & experimental systems

n/a	Involved in the study
☐	☒ Antibodies
☐	☒ Eukaryotic cell lines
☒	☐ Palaeontology and archaeology
☐	☒ Animals and other organisms
☒	☐ Clinical data
☒	☐ Dual use research of concern
☒	☐ Plants

Methods

n/a	Involved in the study
☒	☐ ChIP-seq
☐	☒ Flow cytometry
☒	☐ MRI-based neuroimaging

Antibodies

Antibodies used

The full list is also available in Supplementary Information, Supplementary Table 6.
Antibody Species reactivity Clone Conjugate Cat. # Company
CD16/CD32 (mouse BD Fc blocking) mouse 2.4G2 / 553142 BD
Ter119 mouse TER-119 V450 560504 BD
CD11b mouse M1/70 APC-Cy7 557657 BD
CD45 mouse 30-F11 APC-Cy7 557659 BD
Gr1 mouse RB6-8C5 APC-Cy7 557661 BD
CD44 mouse IM7 APC 559250 BD
CD44 mouse IM7 FITC 553133 BD
Gr-1 mouse RB6-8C5 Biotin 13-5931-75 ebioscience
CD11b mouse M1/70 Biotin 13-0112-75 ebioscience
CD3e mouse 145-2C11 Biotin 13-0031-75 ebioscience
B220 mouse RA3-6B2 Biotin 103204 BioLegend
Ter119 mouse TER-119 Biotin 13-5921-75 ebioscience
CD41 mouse eBioMWReg30 eFluor 450 48-0411-80 Invitrogen
CD16/CD32 mouse 93 BV421 101331 BioLegend
CD34 mouse MEC14.7 APC 119310 BioLegend
Sca-1 mouse D7 APC 108112 BioLegend
CD117 (c-Kit) mouse 2B8 APC-Cy7 105826 BioLegend
Sca-1 mouse D7 BV605 108134 BioLegend
CD71 mouse C2 FITC 553266 BD
GPA human GA-R2 (HIR2) PE 555570 BD
α4-integrin human MZ18-24A9 APC 130-093-281 Miltenyi Biotec
Band3 human / FITC / our lab
CD34 human 8G12 PE 340669 BD
CD36 human CLB-IVC7 FITC 656152 BD
GPA human GA-R2 (HIR2) APC 551336 BD
IL-3R human 6H6 PE-Cy7 570777 BD
CD71 Human M-A712 APC 551374 BD
Ki67 Human/mouse 11F6 FITC 151211 BioLegend
Streptavidin / / PerCP 405213 BioLegend
PIM1 Monoclonal Antibody (ZP003) Human / / 39-4600 Invitrogen
Pim-1 Antibody Human/mouse 12H8 sc-13513 Santa Cruz

Validation

All antibodies were obtained from commercial sources as specified,with the exception of the Band3 antibody,which was generated and rigorously validated in our laboratory. This antibody has been well characterized and extensively used in multiple published studies. For commercially sourced antibodies,only those with vendor-provided validation (including in vitro and in situ assays for IHC/IF/WB,supported by documented evidence on vendor websites) and/or strong literature support were selected. Detailed validation data and references are publicly accessible via the provided catalog numbers. In our experiments, immunofluorescence (IF) staining patterns consistently matched the expected cellular localization reported in prior studies,both before and after DNA strand conjugation.

Eukaryotic cell lines

Policy information about cell lines and Sex and Gender in Research

Cell line source(s)

K562, HEL and HEK 293T cells.

Authentication

Cell lines were not authenticated (not relevant for the experiments or results).

Mycoplasma contamination

All cell lines were confirmed to be mycoplasma free through routine testing.

Commonly misidentified lines
(See ICLAC register)

No commonly misidentified cell lines were used.

Animals and other research organisms

Policy information about studies involving animals; ARRIVE guidelines recommended for reporting animal research, and Sex and Gender in Research

Laboratory animals	Both male and female of EpoR-tdTomato-Cre mice and Pim1fl/flEpoRcre mice at 3-5 month-old were used in this study.
Wild animals	The study did not involve wild animals.
Reporting on sex	This information has not been collected.
Field-collected samples	This study did not involve samples collected from the field.
Ethics oversight	Animal protocols were reviewed and approved by the Animal Ethics Committee of theInstitute of Zhengzhou University.

Note that full information on the approval of the study protocol must also be provided in the manuscript.

Plants

Seed stocks	Not involved.
Novel plant genotypes	Not involved.
Authentication	Not involved.

Flow Cytometry

Plots

Confirm that:

☒ The axis labels state the marker and fluorochrome used (e.g. CD4-FITC).

☒ The axis scales are clearly visible. Include numbers along axes only for bottom left plot of group (a 'group' is an analysis of identical markers).

☒ All plots are contour plots with outliers or pseudocolor plots.

☒ A numerical value for number of cells or percentage (with statistics) is provided.

Methodology

Sample preparation	For human erythropolesis,erythroid progenitors were analyzed by surface markers including IL-3R, GPA, CD34 and CD36, terminal differentiated erythroid cells were analyzed by GPA,Band3 and a4-integrin.For in vivo mouse erythroid progenitors, lineage- cells were enriched and analyzed with surface markers including CD16/32, CD41, CD34, Sca1, c-Kit and CD71; for in vivo mouse terminal erythropolesis, total BM cells were analyzed by CD45, Gr1, CD11b, Ter119 and CD44. for in vitro mouse terminal erythropolesis, cultured cells were analyzed by Ter119 and CD71. Hoechst33342 was used for erythroid enucleation analysis. Annexin V was used for cell apoptosis analysis. Ki67 was used for cell cycle analysis. 7-AAD was used as viability marker.
Instrument	Cells were analyzed within 1 hour of staining using BD FACSDiva Version 6.1.2 software on a LSR Fortessa flow cytometer (Becton Dickinson).
Software	Flow cytometry data were analyzed using FlowJo software.
Cell population abundance	The cell population purity was confirmed to exceed 90% through post-sorting analysis by flow cytometry.
Gating strategy	To analyze human erythroid progenitors, first gate the main cell population using FSC vs SSC to exclude debris and low-complexity events. Next, ensure single-cell analysis by plotting FSC-H vs FSC-A and gating the tight diagonal population to remove doublets. After selecting singlets, apply a viability gate by plotting FSC-H vs 7AAD and excluding 7AAD+ cells (dead/dying cells) while retaining the 7AAD- population (viable cells). From these live single cells, isolate early erythroid progenitors by selecting GPA-IL-3R- cells, which exclude mature erythrocytes (GPA+) and myeloid/lymphoid progenitors (IL-3R+). Finally, analyze CD34 and CD36 expression within this population: BFU-E (CD34+CD36-) and CFU-E (CD34-CD36+).<br>Gating Strategy for Human Erythroid Precursors: Begin by selecting the main cell population using FSC vs SSC, excluding debris and granular cells (e.g., granulocytes). Next, gate single cells using FSC-H vs FSC-A to remove doublets, then exclude

dead/dying cells by selecting 7AAD-cells in an FSC-H vs 7-AAD plot. From the live singlets, isolate erythroid lineage cells by gating GPA+ using a histogram (GPA vs counts) to ensure high specificity. Within the GPA+ population, differentiate erythroblast subsets by plotting Band3 vs a4 integrin: Proerythroblasts (Pro): Band3(-/lo)α4(hi), Basophilic erythroblasts (Baso): Band3(+)a4(hi), Polychromatic erythroblasts (Poly): Band3(++)a4(med) and Orthochromatic erythroblasts (Ortho): Band3(+++)α4(lo/-).

Gating Strategy for Mouse Erythroid Progenitors: After gating the main cell population (FSC vs SSC), single cells (FSC-H vs FSC-A), and live cells (7AAD-), exclude lineage- committed cells by selecting CD16/32-CD41- (plotted against FSC-H to exclude myeloid progenitors and megakaryocytes). Next, gate CD34-Sca1- cells to remove hematopoietic stem/progenitor cells (HSPCs) and non-erythroid lineages. Within this population,identify erythroid progenitors by plotting CD71 (transferrin receptor) vs c-Kit (CD117): BFU-E: CD71(low/-)c-Kit(+) (early progenitors, low CD71 expression); CFU-E: CD71(high)c-Kit(+) (committed precursors,high CD71 but retaining c-Kit).

Gating Strategy for Mouse Terminal Erythropoiesis: First gate the main cell population using FSC vs SSC to exclude debris and low-complexity events. Next, ensure single-cell analysis by plotting FSC-H vs FSC-A and gating the tight diagonal population to remove doublets.After selecting singlets, apply a viability gate by plotting FSC-H vs 7AAD and excluding 7AAD+ cells (dead/dying cells) while retaining the 7AAD- population (viable cells). From the live single cells (7AAD-), exclude immune lineages by gating CD45-Gr1-CD11b-Ter119+ erythroid cells to remove leukocytes and myeloid cells. Within Ter119+ cells,differentiate maturation stages by plotting FSC-A (cell size) vs CD44: Proerythroblasts (Pro): FSC-A(hi)CD44(hi); Basophilic erythroblasts (Baso): FSC-A(med-hi)CD44(med-hi); Polychromatophilic erythroblasts (Poly): FSC-A(med)CD44(med-lo); Orthochromatic erythroblasts (Ortho): FSC-A(lo)CD44(lo); Reticulocytes(Retic): FSC-A(low)CD44(-/low); MatureRBCs: FSC-A(low)CD44(-). For Ki67 staining, we further analyzed the expression level of Ki67 by histograms.

To assess erythroblast enucleation, begin by gating the main cell population using FSC vs SSC to exclude debris and granular cells, followed by FSC-H vs FSC-A to select singlets and exclude doublets. Next, gate 7AAD- cells to isolate live cells. For enucleation analysis,plot FSC-A (cell size) vs Hoechst33342 (DNA dye): nucleated erythroblasts (Pro-E to Ortho-E) appear as Hoechst33342+, while enucleated cells (reticulocytes/RBCs) are Hoechst33342- and typically exhibit smaller size (lower FSC-A).

For apoptosis analysis, start by gating the main cell population using FSC vs.SSC to exclude debris, followed by FSC-H vs FSC-A to select single cells and eliminate doublets. The apoptotic cells are then identified by plotting Annexin V vs 7AAD, where early apoptotic cells appear as Annexin V+7AAD-,late apoptotic/necrotic cells as AnnexinV+7AAD+, and viable cells as Annexin V-7-AAD-. The apoptotic cell population is defined as all Annexin V+ cells (both early and late apoptotic populations).

For in vitro mouse terminal erythroid differentiation analysis, begin by gating the main cell population using FSC vs SSC to exclude debris, followed by FSC-H vs FSC-A to select singlets and exclude doublets. Next, gate 7AAD- cells to isolate live cells. The differentiation stages are then analyzed by plotting CD71 vs Ter119, which reveals the characteristic maturation pattern: early erythroblasts (Ter119medCD71high), intermediate erythroblasts (Ter119highCD71high), and late erythroblasts/ reticulocytes (Ter119highCD71low).

We use FMO or isotype controls to set thresholds for positivity, ensuring accurate discrimination between subsets. This approach minimizes interference from dead cells and aggregates while precisely identifying erythroid progenitor stages.

☒ Tick this box to confirm that a figure exemplifying the gating strategy is provided in the Supplementary Information.
